# The fatty liver disease–causing protein PNPLA3-I148M alters lipid droplet–Golgi dynamics

**DOI:** 10.1073/pnas.2318619121

**Published:** 2024-04-24

**Authors:** David J. Sherman, Lei Liu, Jennifer L. Mamrosh, Jiansong Xie, John Ferbas, Brett Lomenick, Mark S. Ladinsky, Rati Verma, Ingrid C. Rulifson, Raymond J. Deshaies

**Affiliations:** ^a^Amgen Research, Thousand Oaks, CA 91320; ^b^Amgen Research, South San Francisco, CA 94080; ^c^Proteome Exploration Laboratory, Beckman Institute, California Institute of Technology, Pasadena, CA 91125; ^d^Division of Biology and Biological Engineering, California Institute of Technology, Pasadena, CA 91125

**Keywords:** PNPLA3, fatty liver disease, Golgi apparatus, lipid droplet, phosphoinositide

## Abstract

Nonalcoholic fatty liver disease, recently renamed metabolic dysfunction-associated steatotic liver disease (MASLD), is a progressive metabolic disorder that begins with aberrant triglyceride accumulation in the liver and can lead to cirrhosis and cancer. A common variant in the gene *PNPLA3*, encoding the protein PNPLA3-I148M, is the strongest known genetic risk factor for MASLD. Despite its discovery 20 y ago, the function of PNPLA3, and now the role of PNPLA3-I148M, remain unclear. In this study, we sought to dissect the biogenesis of PNPLA3 and PNPLA3-I148M and characterize changes induced by endogenous expression of the disease-causing variant. Contrary to bioinformatic predictions and prior studies with overexpressed proteins, we demonstrate here that PNPLA3 and PNPLA3-I148M are not endoplasmic reticulum-resident transmembrane proteins. To identify their intracellular associations, we generated a paired set of isogenic human hepatoma cells expressing PNPLA3 and PNPLA3-I148M at endogenous levels. Both proteins were enriched in lipid droplet, Golgi, and endosomal fractions. Purified PNPLA3 and PNPLA3-I148M proteins associated with phosphoinositides commonly found in these compartments. Despite a similar fractionation pattern as the wild-type variant, PNPLA3-I148M induced morphological changes in the Golgi apparatus, including increased lipid droplet–Golgi contact sites, which were also observed in I148M-expressing primary human patient hepatocytes. In addition to lipid droplet accumulation, PNPLA3-I148M expression caused significant proteomic and transcriptomic changes that resembled all stages of liver disease. Cumulatively, we validate an endogenous human cellular system for investigating PNPLA3-I148M biology and identify the Golgi apparatus as a central hub of PNPLA3-I148M-driven cellular change.

## Significance

Fatty liver disease affects nearly a quarter of the world’s population and has both environmental and genetic risk factors. A mutation in the gene *PNPLA3* that converts Ile 148 to Met is the strongest known genetic risk factor for developing fatty liver disease. Using a series of techniques to track endogenous PNPLA3 and PNPLA3-I148M biogenesis and localization, we reveal insights into how the mutation changes cellular dynamics. Although previous reports focus on its role on lipid droplets, we reveal that PNPLA3-I148M also functions at the Golgi apparatus, an organelle critical for protein transport into and out of the cell and lipid signaling. PNPLA3-I148M causes altered Golgi morphology and drives changes reminiscent of liver disease.

Metabolic dysfunction-associated steatotic liver disease (MASLD)^*^ is a burgeoning public health threat, estimated to affect approximately one quarter of the world’s population and growing in prevalence similarly to obesity and type 2 diabetes (1, 2). MASLD represents a spectrum of histological changes, starting with fat accumulation in the liver (i.e., steatosis) and, in some individuals, progresses to an inflammatory condition known as metabolic dysfunction-associated steatohepatitis (MASH), followed by cirrhosis, and in some cases hepatocellular carcinoma (HCC). Despite the global threat it poses, the biological underpinnings of the disease are unclear. A major advance in understanding the disease came with the identification of a nonsynonymous variant (rs738409[G]) in the gene *PNPLA3* (patatin-like phospholipase domain-containing protein 3) which is strongly associated with increased hepatic fat (3). This mutation in *PNPLA3* encodes an isoleucine-to-methionine substitution at position 148 (PNPLA3-I148M). The presence of this variant allele increases both the risk and severity across the entire disease spectrum, making it the strongest known genetic determinant of MASLD to date (4–8).

PNPLA3 is a member of a diverse protein family found in organisms ranging from bacteria to humans (9). Receiving its name from patatin, a nonspecific lipid acyl hydrolase found in potatoes, the patatin-like phospholipase (PNPLA) domain contains an α/β/α sandwich fold and a conserved catalytic dyad (Ser-Asp) lipase motif. PNPLA family members vary in their cellular localizations and have biological functions that include host colonization, membrane maintenance, and triglyceride metabolism (9, 10). Despite the significance of PNPLA3-I148M to human health, details about the function of wild-type PNPLA3 and the mechanism(s) by which PNPLA3-I148M causes pathogenesis remain elusive. Whereas deletion or overexpression of the wild-type variant in mice does not cause steatosis, transgenic PNPLA3-I148M overexpression, or knock-in of the mutation in mice challenged with a high-sucrose diet, cause steatosis (11–14). Reducing PNPLA3-I148M protein levels in the liver, including with I148M-specific siRNA, reduces fatty liver disease phenotypes, supporting a specific role for PNPLA3-I148M in driving MASLD (15–17). Purified wild-type PNPLA3 has been shown to possess triglyceride hydrolase or lysophosphatidic acid acyltransferase (LPAAT) activity, with the mutant variant losing the hydrolase activity or showing elevated LPAAT activity (18–23). However, in vivo and cellular data suggest that the conserved enzyme active site is not required for I148M-driven disease. In sum, these data suggest that PNPLA3-I148M can best be described as a neomorph, with the gene product possessing a novel molecular function. However, contrary to this view, in human induced pluripotent stem cells and organoid systems, PNPLA3-I148M elicits an intermediate phenotype between the wild-type and complete *PNPLA3* knockout (24, 25). A model that has been proposed that is consistent with the neomorph hypothesis is that PNPLA3-I148M sequesters CGI-58/ABHD5, a cofactor for the triglyceride hydrolase ATGL/PNPLA2, impairing triglyceride hydrolysis on cytosolic lipid droplets (LDs) and thereby driving steatosis (26, 27). More studies are needed to determine whether this effect is specific to the mutant variant and how this function accounts for the pleiotropic effects caused by PNPLA3-I148M.

PNPLA3 was originally described as a transmembrane protein that can fractionate with cytosolic LDs and intracellular membranes (22, 23, 28, 29). Cellular studies with overexpressed PNPLA3 and PNPLA3-I148M suggested that the proteins localize to LDs, the endoplasmic reticulum (ER), or the cytosol (26, 29). Considering the significant hydrophobicity of the proteins (containing nearly 50% amino acids with hydrophobic uncharged side chains), it is likely that PNPLA3 and PNPLA3-I148M would need to partition into a membrane structure if not bound to a chaperone or LD (vide infra). Residues 42 to 62 have been manually annotated as a type II signal anchor sequence (uniprot.org), with the majority of the PNPLA3 polypeptide predicted to be in the ER lumen (*SI Appendix*, Fig. S1*A*). The ER is a critical compartment for lipid metabolism and LD biogenesis, with known pathways for ER-to-LD protein trafficking (30, 31), making it a plausible localization site for PNPLA3. However, a model in which PNPLA3 is transmembrane (either with a signal anchor sequence or multiple transmembrane helices) is difficult to rationalize because it would require major structural rearrangements to associate with LDs, which are unique organelles in that they contain a core of neutral lipids surrounded by a phospholipid monolayer (30, 32). Therefore, LDs lack a lumenal compartment for the soluble extracytosolic portions of transmembrane proteins. Nevertheless, a transmembrane orientation for PNPLA3 has not been formally disproven, nor have the localizations of the endogenous human wild-type and I148M variants been determined. Disentangling the site(s) of activity of PNPLA3-I148M is important for understanding its function in lipid homeostasis.

Several studies to investigate PNPLA3-I148M function to date have been conducted in mice, yet the mouse and human orthologs differ in their tissue distribution and in their primary sequences and lengths, confounding extrapolations to human protein mechanism and function (8). Given that MASLD is a major emerging public health crisis and PNPLA3-I148M is the single most important human genetic determinant of the disease, we have set out to understand in detail the biochemistry of the wild-type and mutant protein variants and their impact on cellular biology. In this work, we sought to dissect the biogenesis of the human PNPLA3 and PNPLA3-I148M proteins. Using paired human hepatoma cell lines expressing PNPLA3 or PNPLA3-I148M at endogenous levels, we identify the Golgi apparatus as a central node in PNPLA3-I148M-driven cellular change and characterize alterations in LD-Golgi dynamics that translate to primary human hepatocytes.

## Results

### Constitutive Endogenous Expression of PNPLA3-I148M Increases Cellular LD Content.

**Fig. 1.**
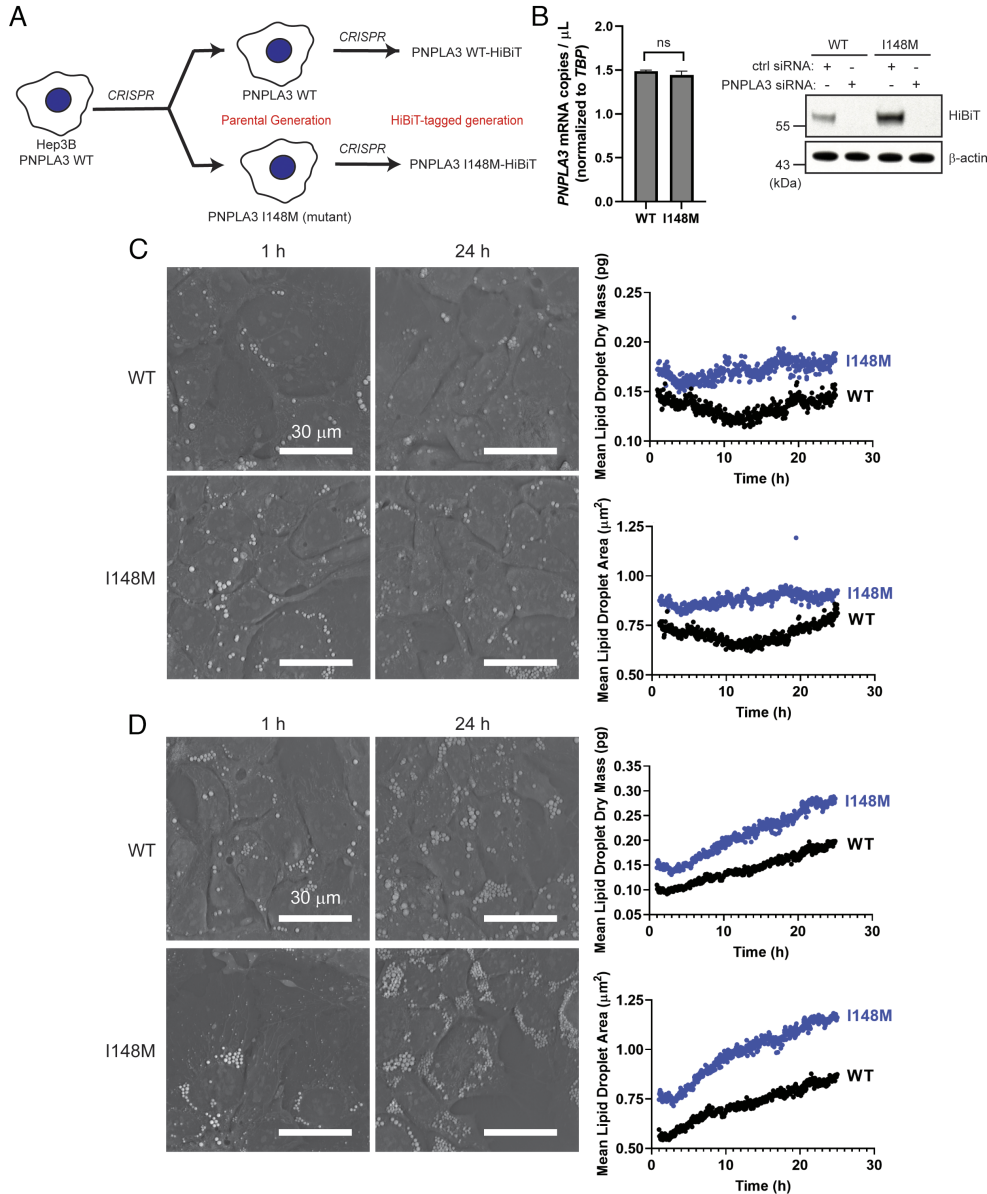
Constitutive endogenous expression of PNPLA3-I148M increases the LD content of hepatoma cells. (*A*) *PNPLA3-I148M* was introduced into Hep3B cells using CRISPR. Parental Hep3B cells (WT and I148M) were further modified to introduce a C-terminal HiBiT tag at the endogenous locus. (*B*) *PNPLA3* expression in the parental cells (*Left*) was analyzed by digital PCR (*P =* 0.0972, determined by Student’s *t* test, n = 3). Protein expression (*Right*) was analyzed by HiBiT detection (bioluminescence observed in the presence of LgBiT protein, as described in *SI Appendix*) in the tagged cell lines treated with a negative control siRNA or PNPLA3-specific siRNA. (*C* and *D*) Representative microscope images (from 1 and 24 h) and quantification of mean lipid droplet content and mean lipid droplet area from live-cell label-free imaging using the Nanolive 3D Cell Explorer microscope. Cells were treated with vehicle (*C*) or with 200 µM oleic acid (*D*). Images were taken every 3 min for 24 h, starting 1 h after addition of oleic acid. Quantification was done using Eve software. Experiment was repeated twice with similar results.

To investigate the biogenesis and cellular effects of endogenously expressed human PNPLA3 and PNPLA3-I148M, we generated a paired set of Hep3B cells using CRISPR. Hep3B is a hepatoma cell line that expresses endogenous wild-type PNPLA3, whereas several typical human hepatoma cell lines express endogenous PNPLA3-I148M. We gene-edited the native *PNPLA3* locus in Hep3B cells to make a homozygous PNPLA3-I148M (I148M) cell line (Fig. 1*A*). Because PNPLA3 is a low-abundance protein and we have been unable to track the endogenous gene product with existing antibodies, we introduced a HiBiT tag at the C-terminus of PNPLA3 in both WT and I148M cells (33, 34). The HiBiT tag complements a nonnatural protein, LgBiT, to form an active enzyme that, in the presence of a substrate, generates luminescence. We confirmed homozygous tagging in the WT and I148M cells. In Hep3B knock-in cells, mRNA levels of PNPLA3 and PNPLA3-I148M were equal, yet the steady-state level of PNPLA3-I148M was ~3 to 4 times greater than that of the WT protein (Fig. 1*B*). This was consistent across multiple HiBiT cell clones and recapitulates what has been reported in mouse models (14).

To evaluate the impact of PNPLA3-I148M expression on the LD content of hepatoma cells, we performed live-cell, label-free imaging using Nanolive, a 3D holotomographic microscopic platform that allows for LD identification by their high refractive index values (35, 36). Even under basal conditions, I148M cells had ~1.4 times greater mean LD dry mass and area than WT cells (Fig. 1*C* and Movies S1 and S2). When cells were treated with oleic acid, there was a steady increase in LD content throughout a 24-h time course, with at least ~1.6 times greater mean LD dry mass and area in I148M cells than in WT cells (Fig. 1*D* and Movies S3 and S4). Mean LD dry mass and area increased in I148M cells at ~1.3 times the rate of WT cells. This mirrors reports that expression of PNPLA3-I148M is enough to drive increases in LD content in the mouse liver and in overexpression cell lines (13, 14, 26, 37).

### PNPLA3-I148M Fractionates with the Golgi and Alters Golgi Structure.

PNPLA3 contains several stretches of hydrophobic amino acids that are of the right length to span a membrane, three of which are in the PNPLA domain (*SI Appendix*, Fig. S1*A*). If PNPLA3 is in fact an integral membrane protein of the secretory or endocytic pathway(s), then it must initiate biogenesis at the ER and contain an ER-targeting signal sequence. To investigate ER membrane targeting and association, we employed a biochemical system to probe the process in vitro. Human PNPLA3 and PNPLA3-I148M with C-terminal hemagglutinin (HA) epitope tags were translated in rabbit reticulocyte lysate in the presence of ^35^S-methionine. In some reactions, canine rough microsomal membranes (cRMs) were included (cotranslationally) or were added after termination of translation (posttranslationally). cRMs were isolated by centrifugation, washed with sodium carbonate to remove peripherally associated proteins, and reisolated (*SI Appendix*, Fig. S1*B*). The majorities of PNPLA3-HA and PNPLA3-I148M-HA were found in the supernatant fraction, with ~10% of the wild-type protein and <5% of the I148M variant found stably inserted in the cRMs posttranslationally (*SI Appendix*, Fig. S1*C*). In contrast, a construct with a deletion of the hydrophobic stretch of amino acids 42 to 62 was not bound to cRMs. Due to the small fraction of PNPLA3-HA stably associated with cRMs, the lack of association of the Δ42 to 62 construct does not provide conclusive evidence that amino acids 42 to 62 constitute a signal anchor sequence. Membrane-associated PNPLA3-HA and PNPLA3-I148M-HA were completely digested by Proteinase K (*SI Appendix*, Fig. S2*A*), inconsistent with the prediction that PNPLA3 contains a significant protease-protected, ER-lumenal C-terminal domain (*SI Appendix*, Fig. S1*A*). This lack of protease protection was also true for microsomes isolated from HEK293T cells expressing PNPLA3 (*SI Appendix*, Fig. S2*B*). Although the protease protection experiments suggest that PNPLA3-HA does not contain an ER-lumenal portion, we tested the hypothesis that parts of PNPLA3 could be inside the ER by treating detergent-solubilized cRMs with Endoglycosidase H (Endo H), which cleaves asparagine-linked mannose-rich oligosaccharides that would be added to PNPLA3 if its consensus N-linked glycosylation sites were inside the microsomal lumen. PNPLA3-HA and PNPLA3-I148M-HA did not show gel migration shifts by sodium dodecyl sulfate-polyacrylamide gel electrophoresis when digested with Endo H, nor were the N and C termini glycosylated when an opsin tag containing a consensus glycosylation site was added (*SI Appendix*, Fig. S2*C*).

If PNPLA3 contains an ER-specific signal sequence (even if not a transmembrane signal anchor), there is conserved ER-specific protein machinery that would be required to recognize and insert it into the membrane (38). To determine whether protein insertases at the ER are required for stable association of PNPLA3-HA or PNPLA3-I148M-HA in this biochemical system, we posttranslationally added cRMs that were trypsin-treated (“T-cRMs”) or “mock” treated (“M-cRMs”) to in vitro translation reactions (39, 40). We hypothesized that if membrane insertion relied on traditional pathways at the ER, digesting the exposed membrane proteins with trypsin would eliminate protein insertion. We found that PNPLA3-HA and PNPLA3-I148M-HA could still be inserted into T-cRMs (*SI Appendix*, Fig. S2*D*). Taken together, these experiments suggest that PNPLA3 and, to a lesser extent, PNPLA3-I148M, can insert into ER-derived membranes without membrane-resident protein insertion machinery, and the portion of each protein stably inserted into the membrane is small and does not traverse the phospholipid bilayer. This is inconsistent with traditional ER transmembrane proteins but consistent with traditional LD proteins, which often form membrane hairpins that allow for stable insertion into the outer phospholipid monolayer (41).

**Fig. 2.**
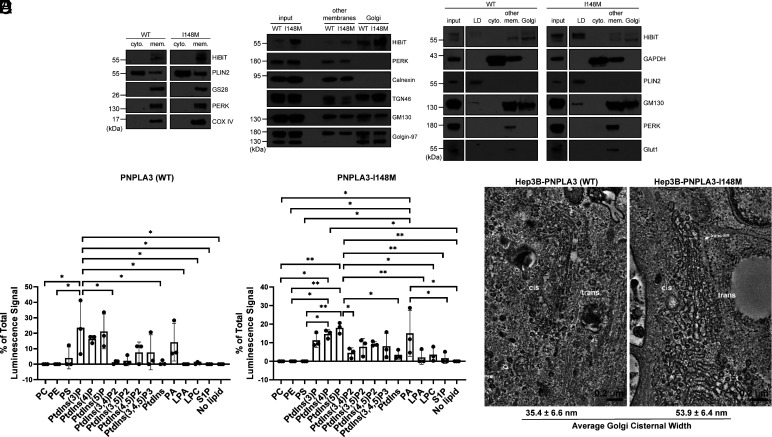
PNPLA3-I148M induces structural changes at the Golgi apparatus. (*A*) Basal-state Hep3B cells expressing endogenous PNPLA3-HiBiT or PNPLA3-I148M-HiBiT were fractionated into cytosolic (“cyto.”) or total membrane (“mem.”) fractions. (*B*) Basal-state Hep3B-HiBiT cells were further fractionated to separate membrane species. Other membranes include non-Golgi membranes. (*C*) Hep3B-HiBiT cells were treated with 100 µM oleic acid for 16 h prior to fractionation into LD, cytosolic, other membranes, and Golgi membrane fractions. (*D*) Purified PNPLA3-His and PNPLA3-I148M-His were incubated with phosphoinositide strips containing spots with 100 pmol of different lipid species. Following washing and staining with anti-His-HRP, strips were imaged using chemiluminescence. Bar graph represents the average of three independent experiments. *P*-values were calculated from an ordinary one-way ANOVA with Tukey’s multiple comparisons test. (*E*) Hep3B cells (WT or I148M) were treated with 100 µM oleic acid for 16 h, then fixed and imaged by TEM. Average cisternal widths were calculated from 20 Golgi cisternae per cell type. Fractionation studies (*A*–*C*) were repeated 2 to 4 times with similar results. TEM (*E*) was repeated twice. *P < 0.05; **P < 0.01.

Next, we sought to determine where PNPLA3 and PNPLA3-I148M localize in the endogenous Hep3B cellular system. We fractionated the postnuclear supernatant of basal-state Hep3B cells into cytosolic and crude membrane fractions. PNPLA3-HiBiT and PNPLA3-I148M-HiBiT proteins isolated entirely with crude membranes (Fig. 2*A*). This differed from perilipin-2 (PLIN2), the majority of which remained in the cytosolic fraction, consistent with its designation as a type II LD protein that is targeted to LDs from the cytosol (32). We next determined the specific membrane fraction(s) with which PNPLA3 and PNPLA3-I148M isolate. Of note, ~78% of PNPLA3-HiBiT and ~69% of PNPLA3-I148M-HiBiT were isolated with the Golgi membranes, with the remaining protein pools found in the “other membranes” fraction, which included the ER and plasma membrane (Fig. 2*B*). Similar results were obtained fractionating another pair of HiBiT cell clones.

When Hep3B cells were treated with oleic acid overnight to stimulate LD formation, ~15% of the WT protein and ~72% of I148M redistributed to the buoyant LD fractions (Fig. 2*C*), whereas ~61% and ~18% of the WT and I148M proteins, respectively, were found in the enriched Golgi fractions. The remaining protein pools were isolated with other membranes. In a separate experiment, PNPLA3-HiBiT and PNPLA3-I148M-HiBiT were found in endosomes isolated from basal-state Hep3B cells (*SI Appendix*, Fig. S3*A*). While we cannot determine whether the endosomal pool is derived from the other membrane or Golgi fractions, and how this changes with oleic acid stimulation, it is notable that other LD proteins, including Rab GTPases, can also localize to endosomes (42).

To confirm colocalization of PNPLA3-HiBiT and PNPLA3-I148M-HiBiT with the Golgi in live cells, we generated HiBiT-tagged cell lines coexpressing the LgBiT protein. In the presence of a cell-permeable substrate, the high-affinity HiBiT-LgBiT complex generates bioluminescence. Using these HiBiT-LgBiT cell lines, we performed live cell bioluminescence imaging, combined with fluorescence imaging of BODIPY FL C_5_-ceramide, which preferentially stains Golgi membranes. We observed colocalization of the PNPLA3-derived bioluminescence signal and the fluorescence signal in both WT and I148M cell lines (*SI Appendix*, Fig. S4).

To determine how PNPLA3 and PNPLA3-I148M might associate with the secretory/endosomal pathway, we tested whether the purified proteins bind to membrane and signaling lipids commonly found in the Golgi, endosome, and plasma membrane. Purified PNPLA3 and PNPLA3-I148M bound to lipid arrays spotted with different phosphoinositide species, especially PtdIns(3)P, PtdIns(4)P, and PtdIns(5)P (Fig. 2*D* and *SI Appendix*, Fig. S3 *B* and *C*). These phosphoinositides are enriched in the Golgi/*trans*-Golgi network and in the endosomal system (43). PNPLA3-I148M demonstrated even greater selectivity for phosphoinositide species from membrane phospholipids than the WT protein. Otherwise, there were no significant differences in the lipids bound by the WT and mutant variants. In addition to phosphoinositide binding, both proteins interacted with phosphatidic acid (Fig. 2*D*). Similar results were observed using proteins purified from whole cell lysate or from the membrane fraction, with either C-terminal His- or FLAG-tags. To confirm the quality of the lipid-binding arrays, we verified that purified GST-tagged PLC-δ1 PH domain protein correctly interacted with PtdIns(4, 5)P2 (*SI Appendix*, Fig. S3*C*) (44, 45).

Because PNPLA3-I148M can bind phospholipids important for vesicular trafficking, signaling, and membrane dynamics, we investigated whether there were morphological changes in I148M cells. Hep3B cells (WT and I148M) were treated with oleic acid, fixed, and imaged using transmission electron microscopy (TEM). Whereas the ER was similar in both cell lines, the I148M cells demonstrated marked enlargement of Golgi cisternae (Fig. 2*E*). These enlarged structures had lucent interiors and could be observed among the *cis*-, *medial*-, and *trans*-Golgi network.

### Endogenous PNPLA3-I148M Alters the Proteomic and Transcriptomic Landscape of Hep3B Cells.

**Fig. 3.**
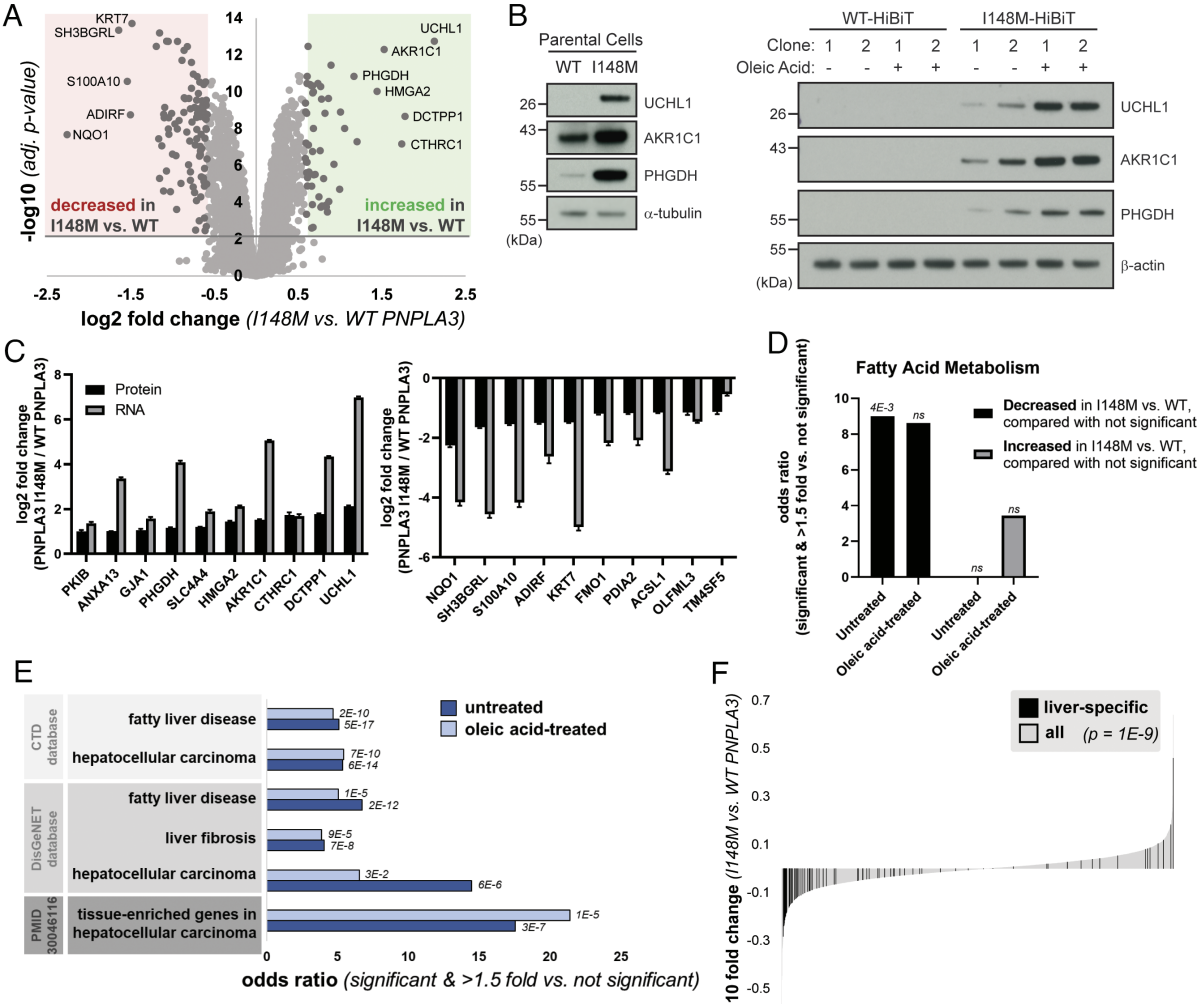
PNPLA3-I148M causes proteomic changes consistent with liver disease. (*A*) Volcano plot of proteins quantified by mass spectrometry in untreated cells expressing WT or PNPLA3-I148M. Red and green compartments contain proteins significantly (adj. *P*-value < 0.01) decreased or increased, respectively, by >1.5 fold in the I148M vs. WT cell lines. (*B*) Confirmation of top proteomic hit proteins in parental Hep3B cells and additional HiBiT clones, with and without incubation with 200 µM oleic acid overnight. (*C*) Log2 fold changes of top 10 significantly upregulated (*Left*) and significantly downregulated (*Right*) proteins in untreated mass spectrometry, compared with gene expression from RNA-seq. (*D*) Enrichment of the biological process keyword in proteins significantly (adj. *P*-value < 0.01) decreased or increased by >1.5 fold in the I148M vs. WT PNPLA3 cell lines vs. those not significantly changing. Fatty acid metabolism is the only term that is significant after multiple testing correction in at least one comparison. *P*-values indicated above the bars. (*E*) Proteins significantly (adj. *P*-value < 0.01) decreased or increased by >1.5 fold in the I148M vs. WT PNPLA3 cell lines were compared with those not significantly changed. Enrichment of liver disease-related terms was determined; adjusted *P*-value by Fisher’s exact test is shown. (*F*) Plot showing the log10 fold change for proteins quantified in the I148M vs. WT PNPLA3 untreated cell lines. Those reported (TiGER database) to be liver-specific genes are shown in black. The Kolmogorov–Smirnov *P*-value testing for a difference in fold change distribution between the two protein sets is reported.

As the nexus of the secretory and endolysosomal pathways, changes in Golgi architecture can affect fundamental cellular processes. We were interested in whether constitutive expression of endogenous PNPLA3-I148M resulted in proteomic changes in the cell. Therefore, we conducted quantitative mass spectrometry on cell lysates of WT- and I148M-expressing Hep3B-HiBiT cells treated with or without oleic acid (Fig. 3*A* and *SI Appendix*, Fig. S5*A*). We observed that I148M-expressing cells had both significantly increased and decreased levels of multiple proteins compared to WT cells. The effect of the PNPLA3 variant on protein abundance levels was similar between untreated cells and cells treated with oleic acid for 16 h, suggesting that the I148M variant has a distinct effect on Hep3B cells compared to oleic acid treatment (*SI Appendix*, Fig. S5*B*). To ensure that these changes were not due to clonal effects or the HiBiT tag, we confirmed top changing proteins in parental cells and other HiBiT cell clones (Fig. 3*B* and *SI Appendix*, Fig. S5*C*).

We also used mass spectrometry to quantify phosphorylation of peptides in WT- and I148M-expressing Hep3B-HiBiT cells treated with or without oleic acid. Similar to what we observed by whole-cell proteomics, oleic acid treatment had a relatively minor impact on phosphorylated peptide levels (*SI Appendix*, Fig. S5*D*). We considered that phosphorylated protein abundances that significantly differed between I148M and WT cells could be due to differential protein phosphorylation, or differential protein expression levels. Comparison of the phosphoproteomics and whole-cell proteomics datasets suggested that many proteins differentially phosphorylated between cell lines could be predicted by protein abundance alone; however, a smaller number of proteins appeared to be differentially phosphorylated (*SI Appendix*, Fig. S5*E*). One of these proteins, TANGO1, stood out because it functions in the secretory pathway and is required for the export of bulky collagen and large lipid particles from the ER (46, 47). TANGO1 is not significantly changed at the protein level in PNPLA3-I148M cells, but one residue, S358, is significantly less phosphorylated in I148M cells compared to WT cells.

In addition to proteomics, we performed RNA sequencing (RNA-seq) on these cell lines. When comparing the proteomics and RNA-seq datasets from untreated cells, the top changing proteins in the proteomics datasets were also changing in the corresponding direction at the mRNA level (Fig. 3*C*). Therefore, these proteomic changes were due, at least in part, to transcriptional effects. We considered whether there were differences in biological function between proteins significantly changed in the I148M vs. the WT cells, as compared with proteins not significantly changed. Proteins decreased in untreated I148M cells were more likely to be involved in fatty acid metabolism, consistent with a putative function of PNPLA3 in lipid homeostasis (Fig. 3*D*).

We also observed that proteins significantly changed in I148M vs. WT cells were more likely to overlap with genes associated with fatty liver disease, liver fibrosis, and HCC (Fig. 3*E* and *SI Appendix*, Fig. S5*F*). Furthermore, liver-specific genes were enriched in proteins significantly changed in these cell lines (Fig. 3*F*). These genes, the majority of which were downregulated in I148M, reflect a “dedifferentiation” of hepatocytes observed in fatty liver disease and HCC (48, 49). Indeed, proteins decreased in the I148M-expressing cells were more likely to be liver-specific genes that are also downregulated in HCC (*SI Appendix*, Fig. S5*G*). Therefore PNPLA3-I148M, even in the absence of exogenous lipid stimulation, is sufficient to promote an effect resembling dedifferentiation of hepatocytes associated with the development of HCC, even when evaluated in hepatoma cell lines.

### Endogenous PNPLA3-I148M Increases LD-Golgi Contact Sites in Primary Human Hepatocytes.

**Fig. 4.**
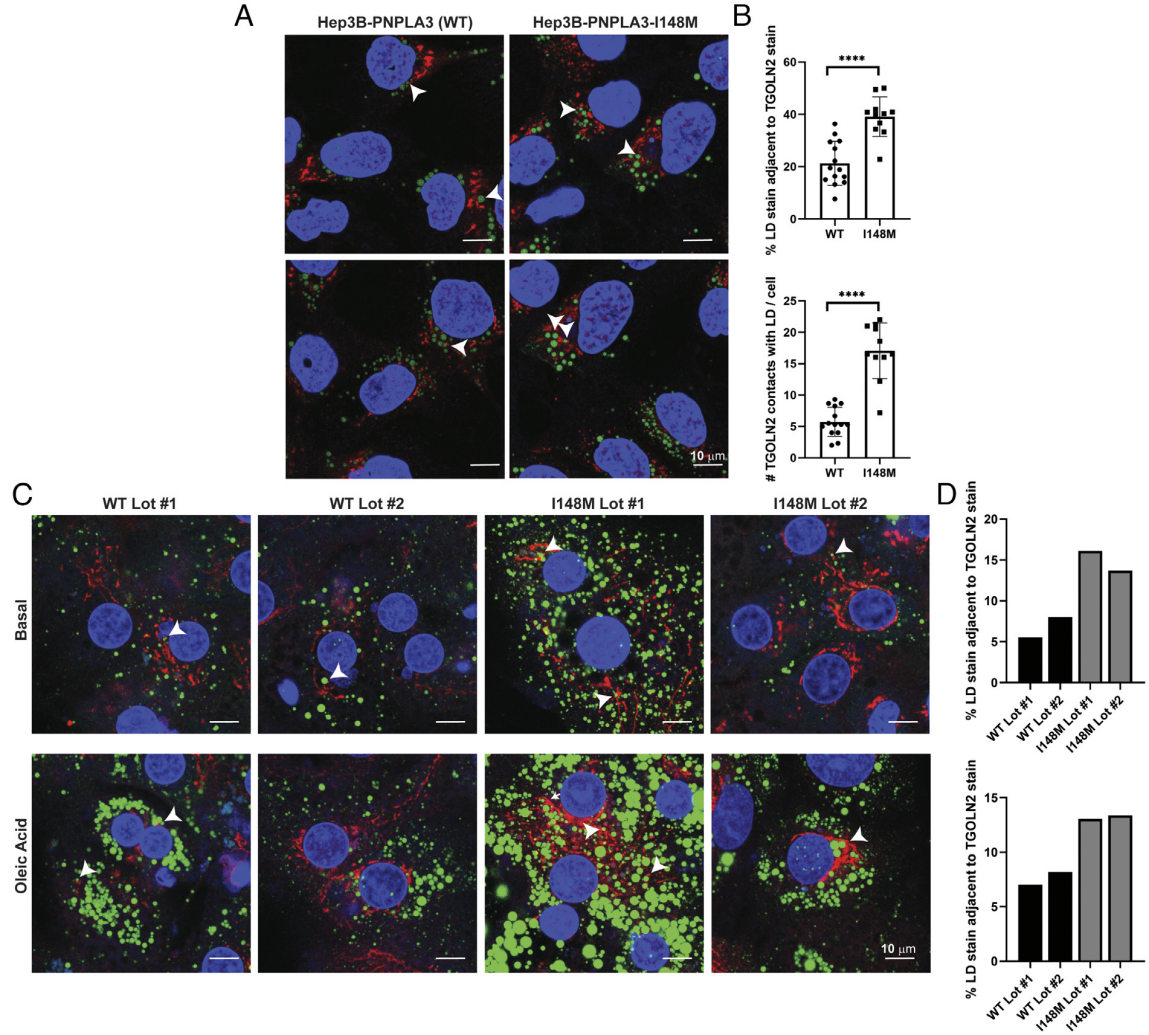
Hepatoma cells and primary hepatocytes expressing PNPLA3-I148M have more LD-Golgi contacts. (*A*) Hep3B cells expressing endogenous PNPLA3 or PNPLA3-I148M were fixed and stained with LipidTOX Green to label LDs (green) and anti-TGOLN2 (red) to label the *trans*-Golgi network. White arrowheads indicate examples of LD-TGOLN2 interactions. (*B*) Lipid droplet-TGOLN2 contact sites in ~45 cells per cell line were quantified using Imaris software. *P-*values were calculated from Student’s *t* tests. (*C*) Individual lots of primary human hepatocytes expressing wild-type PNPLA3 or PNPLA3-I148M were plated, treated with vehicle or 200 µM oleic acid for 24 h, fixed and stained with LipidTOX Green to label LDs (green) and anti-TGOLN2 (red) to label the *trans*-Golgi network. White arrowheads indicate examples of LD-TGOLN2 interactions. (*D*) 20 to 40 cells of each lot and condition were quantified and LD-TGOLN2 proximity was calculated using Imaris software. Graphs display percentage of LD stain juxtaposed to TGOLN2 stain for untreated cells (*Top*) and oleic acid-treated cells (*Bottom*) per primary hepatocyte lot. WT (black) and I148M (gray) lots are indicated. Each bar represents the average of 11 fields of view for each primary hepatocyte lot per condition. ****P < 0.0001.

Hep3B cells expressing PNPLA3-I148M have greater LD content (Fig. 1 *C* and *D*), and qualitatively, there appeared to be more LDs in the vicinity of the Golgi compared to WT cells, as judged by TEM imaging (*SI Appendix*, Fig. S6 *A* and *B*). To test whether there were changes in LD-Golgi contacts, Hep3B cells were fixed, permeabilized, and stained with anti-TGOLN2 (Golgi; red stain) and LipidTOX Green (LD; green stain) for visualization by confocal microscopy (Fig. 4*A*). After separately quantifying the LD and TGOLN2 staining areas using a machine learning approach, LD-TGOLN2 interactions were quantified by counting juxtaposed staining sites. Staining sites within 30 nm of each other were considered juxtaposed based on an acceptable cutoff for determining membrane contact sites (50). Consistent with the TEM imaging, mutant Hep3B cells had a greater percentage of LD stain adjacent to TGOLN2, and vice versa (Fig. 4*B*). Additionally, we qualitatively observed a more dispersed TGOLN2 signal in I148M cells. We only observed this change in LD-TGOLN2 juxtaposition under basal conditions, as oleic acid treatment appeared to mask the phenotype (*SI Appendix*, Fig. S6*C*).

Considering the disease-like changes induced by endogenous PNPLA3-I148M in the hepatoma cells, we asked whether endogenous expression of PNPLA3-I148M can induce similar changes in LD-Golgi dynamics in primary human hepatocytes. Primary hepatocyte lots (each lot derived from a different patient) expressing either WT PNPLA3 or homozygous for rs738409[G] were identified (*SI Appendix*, Fig. S7). The hepatocytes were either maintained in media lacking fatty acid supplementation or treated with 200 µM oleic acid for 24 h. Then, the cells were fixed, permeabilized, and stained with anti-TGOLN2 and LipidTOX Green (Fig. 4*C*). As in Hep3B cells, contact sites were quantified. Despite significant differences in LD and Golgi content between lots (even between the two PNPLA3-I148M-expressing lots), there were overall more LD-TGOLN2 contact sites in the mutant hepatocytes compared to the WT hepatocytes (Fig. 4*D*). Unlike with Hep3B cells, we observed this phenotype in both untreated and oleic acid-treated cells. This suggests that rs738409[G] is driving the LD-TGOLN2 contact trends despite the genetic variability in these four lots.

## Discussion

In this study, we investigated the biogenesis of PNPLA3 and PNPLA3-I148M and the cellular consequences of constitutive endogenous I148M expression in hepatoma cells. We generated a paired set of Hep3B cells expressing PNPLA3-HiBiT and PNPLA3-I148M-HiBiT and demonstrated that relative PNPLA3 abundance and cellular LD content recapitulate prior reports, including studies with overexpression cell lines and knock-in mice (Fig. 1) (14). Then, we sought to resolve conflicting reports on the nature of PNPLA3 localization and topology. Using an in vitro translation system, we could not establish that PNPLA3 is a transmembrane protein nor that it is targeted to the ER with a signal sequence (*SI Appendix*, Figs. S1 and S2). Using a highly sensitive luminescent reporter tag, we demonstrated that endogenous PNPLA3 and PNPLA3-I148M isolate with cellular LD, Golgi, and endosomal fractions (Fig. 2). As a basis for such localization, we interrogated lipid binding properties of the purified WT and I148M proteins and found that they interact with phosphoinositides enriched in Golgi and endosomal membranes. Through imaging studies, we identified distinct morphological changes in the Golgi apparatus in I148M-expressing cells (Fig. 2 and *SI Appendix*, Fig. S6 *A* and *B*). To complement the morphological changes, whole-cell proteomics, phosphoproteomics, and RNA-seq revealed altered proteomic and transcriptomic profiles in I148M-expressing cells that are consistent with those found in the full spectrum of liver disease, supporting a role for I148M in driving all stages of MASLD (Fig. 3 and *SI Appendix*, Fig. S5). Finally, more LD-Golgi contacts were observed in Hep3B cells and primary human hepatocytes expressing endogenous PNPLA3-I148M (Fig. 4), suggesting a role for PNPLA3-I148M, either directly or indirectly, in restructuring this central hub of the secretory pathway and its interactome.

Dissecting the differences between WT PNPLA3 and PNPLA3-I148M can greatly enhance our understanding of the genetic basis for MASLD and fundamental lipid metabolism. As such, this work can help to refine our grasp of what “goes awry” with PNPLA3-I148M. The finding that PNPLA3 and PNPLA3-I148M are more enriched in the Golgi than the ER highlights the importance of the Golgi in lipid homeostasis and human disease. Many lipid metabolic enzymes localize to the ER (30, 51). The Golgi, though important for hepatic very-low-density lipoprotein biogenesis (52–54), has not been extensively investigated as a meaningful node for MASLD/MASH nor for PNPLA3 biology. As a bustling depot for intracellular cargo intimately integrated with the cytoskeleton, changes in the Golgi apparatus can have pleiotropic effects on signal transduction, lipid trafficking, and membrane homeostasis (55, 56). One study found that loss-of-function MASLD genetic risk factors introduced into HepG2 hepatoma cells, which express endogenous PNPLA3-I148M, caused enlarged Golgi cisternae relative to parental cells, but this study did not investigate the effects of PNPLA3-I148M alone on the Golgi (57). Lending credence to the findings presented here, an RNAi screen in *Drosophila* S2 cells identified members of the COPI vesicular trafficking machinery, which facilitate retrograde Golgi-to-ER and intra-Golgi vesicular transport, as significant regulatory factors for LD formation and morphology (58). Additionally, liver proteomics and phosphoproteomics in a diet-induced MASLD mouse model demonstrated reorganization of the secretory pathway, including redistribution of COPI proteins and segregation of Golgi proteins to LDs (59). Although we have established an association of PNPLA3-I148M with the Golgi, future work is needed to investigate the determinants of localization, considering that there are no known pathways for Golgi-to-LD protein trafficking.

Membrane contact sites have gained increasing attention as important nodes of interorganelle communication and sites of ion, lipid, and metabolite transfer (50). A systems-level characterization of organelle contact sites in COS-7 cells identified LD interactions with most other organelle types, with Golgi contacts representing the second-most abundant heterotypic LD interaction (~15% of LDs contacted the Golgi) (60). Although LD-Golgi interactions are clearly significant and potentially important in fatty liver disease (59, 61), these contact sites are poorly characterized, in part due to the unique shape and composition of the Golgi and the diversity of Golgi cisternae within a single cell (62). Although we found closer apposition of LDs and the Golgi in I148M cells compared to WT cells, the nature of these interactions, including whether they are directly mediated by PNPLA3-I148M, is the topic of future work. Limitations in antibodies suitable for endogenous PNPLA3 immunofluorescence have made such detailed studies challenging to conduct thus far.

Another notable finding from this work is that PNPLA3 and PNPLA3-I148M can associate with membrane phosphoinositides despite lacking an annotated phosphoinositide-binding domain, such as the pleckstrin homology (PH) or FYVE domain (43). Phosphoinositides are important for regulating membrane dynamics and maintaining organellar identity, especially within the secretory and endolysosomal pathways (43). Phosphoinositides and phosphoinositide-binding proteins also facilitate organellar junctions and nonvesicular lipid trafficking between cellular compartments, exemplified by the role of oxysterol binding protein in coupling sterol trafficking from the ER to the Golgi with PtdIns(4)P transport from the Golgi to the ER, leading to its hydrolysis (63). An association between PNPLA proteins and phosphoinositides is not unprecedented, as host cell plasma membrane localization of the *Pseudomonas aeruginosa* virulence factor ExoU, a PNPLA family member, is mediated by its association with PtdIns(4, 5)P2 (64). Future work will uncover the nature of the PNPLA3-phosphoinositide interaction and the role that these lipids play in PNPLA3 biogenesis and function.

The knock-in cell lines developed in this study can be used to delve deeper into PNPLA3 biology. Although there are limitations to using cell lines to study the role of proteins in human disease, the key differences between mouse and human PNPLA3 tissue distribution and primary sequence, coupled with technical limitations and heterogeneity of primary human hepatocytes, prompted us to develop a controlled hepatoma cell system to directly compare the WT and I148M proteins. The fact that PNPLA3-I148M drives the full spectrum of MASLD, including HCC, further supports use of this cellular system. One published study used genetically engineered human induced pluripotent stem cells to model PNPLA3-I148M function with similar reasoning to ours (24). However, the authors concluded that *PNPLA3-I148M* is a loss-of-function mutation rather than neomorphic, the former of which is inconsistent with both mouse and human data pointing to PNPLA3-I148M protein as a driver of MASLD. The characteristic disease-like changes we observed in Hep3B-PNPLA3-I148M cells bolster our ability to extrapolate findings to the disease state. Accordingly, we confirmed our observation of altered LD-Golgi dynamics in primary human PNPLA3-I148M hepatocytes.

Introducing a single amino acid change into PNPLA3 can change the morphological, proteomic, and transcriptomic landscape of the cell. Lipid metabolic pathways are tightly integrated and regulated, and the downstream effects of PNPLA3-I148M on the cellular proteome are pleiotropic, much like MASLD. Unraveling the causes and effects of the phenotypes and identifying the most upstream function of PNPLA3-I148M, starting with its initial biogenesis, could greatly enhance our understanding of lipid homeostasis and lead to the development of impactful therapeutics to curb the silent epidemic that is MASLD.

## Materials and Methods

### Cell Lines.

Hep3B cells (ATCC) were grown in Eagle’s Minimum Essential Medium (EMEM; ATCC) supplemented with 10% dialyzed fetal bovine serum (Gibco) and 1x Antibiotic-Antimycotic (Gibco). Details on gene editing are provided in *SI Appendix*.

For siRNA treatment, Silencer Select Negative Control #1 (Invitrogen 4390843) and Silencer Select PNPLA3 (Invitrogen s37253) siRNA reagents were used. Cells were reverse-transfected using Lipofectamine RNAiMAX (Invitrogen), following the manufacturer’s protocol, and treated for 72 h.

### Label-Free Imaging for Lipid Droplet Dynamics.

Hep3B cells were seeded into 96-well glass-bottom plates (MatTek Corporation) at a density of ~30,000 cells per 80 µL of phenol red-free EMEM (Quality Biological) and incubated overnight at 37 °C. Then, prior to imaging, media was exchanged for fresh phenol red-free media with or without oleic acid-BSA (Sigma). Plates were briefly centrifuged to prevent meniscus formation and placed on the pre-equilibrated (37 °C, 5% CO_2_ with humidity) Nanolive CX-A 3D Cell Explorer microscope (Nanolive). After approximately 1 h, thermalization was complete, and time-lapse refractive index images were captured every 3 min for 24 h using 3 × 3 gridscan mode (a field view of 275 × 275 µm). Images were segmented and quantified using the Smart Lipid Droplet Assay ^LIVE^ Module in the Eve software (Version 1.9.2.1760; Nanolive).

### Cellular Fractionations.

Crude membranes and LDs were isolated as described (65) or by commercial precipitation-based methods to enrich for organelle subfractions (Invent Biotechnologies).

### Immunofluorescence.

Hep3B cells were grown in Nunc Lab-Tek Chambered Slides (ThermoFisher Scientific). Primary human hepatocytes were grown in Collagen I-coated chambered slides (Ibidi). Cells were washed and fixed in 4% formaldehyde (Sigma), as previously described (66). Fixed cells were washed twice with PBS and incubated in 50 mM ammonium chloride (Sigma) for 10 min at room temperature. After two PBS washes, samples were permeabilized in 0.1% Triton X-100 for 10 min at room temperature. Fixed and permeabilized cells were washed twice in PBS and blocked in 5% normal goat serum (Gibco)/1% BSA for 30 min at room temperature. Primary antibodies (*SI Appendix*, Table S3) were added in 1% BSA, and chamber slides kept at 4 °C overnight. Samples were washed in PBS (3 × 5 min at room temperature) and then incubated with secondary antibodies diluted in 1% BSA for 1 h at room temperature (in the dark). Following PBS washes (3 × 5 min at room temperature), samples were incubated with 2 µM Hoechst 33342 (ThermoFisher Scientific) and HCS LipidTOX Green Neutral Lipid Stain (ThermoFisher Scientific) for 30 min at room temperature. The solution was exchanged for PBS, and images were obtained on a SP8 confocal microscope (Leica) using a 63× glycerol immersion lens.

### Juxtaposition Analysis.

Raw confocal images were analyzed in Fiji (67), where LD and TGOLN2 signals were separately segmented using machine learning pixel classification from Labkit (68). Segmentation data were imported as surfaces into Imaris 10.0.0 (Oxford Instruments) and juxtaposition analysis was performed by measuring the distance between surfaces (surfaces within 30 nm of each other were considered juxtaposed).

## Supplementary Material

Appendix 01 (PDF)

Dataset S01 (XLSX)

Dataset S02 (XLSX)

Dataset S03 (XLSX)

Movie S1. Nanolive time course for untreated Hep3B PNPLA3 (WT) cells.

Movie S2. Nanolive time course for untreated Hep3B PNPLA3-I148M cells.

Movie S3. Nanolive time course for oleic acid-treated Hep3B PNPLA3 (WT) cells.

Movie S4. Nanolive time course for oleic acid-treated Hep3B PNPLA3-I148M cells.

## Data Availability

Mass spectrometry-based proteomics RNA-seq data have been deposited in PRIDE (for Proteomics) (69) and GEO (for RNA-seq) (70) [PXD046335 (for PRIDE) GSE261297 (for GEO)].

## Footnotes

^*^As of June 2023, the American Association for the Study of Liver Diseases (AASLD) has renamed nonalcoholic fatty liver disease (NAFLD) as MASLD and nonalcoholic steatohepatitis as MASH (https://www.aasld.org/new-nafld-nomenclature).
