# Supplementary material for: The fatty liver disease–causing protein PNPLA3-I148M alters lipid droplet–Golgi dynamics
Source: Proc Natl Acad Sci U S A. 2024 Apr 24;121(18):e2318619121. doi: 10.1073/pnas.2318619121 (PMC11067037; doi:10.1073/pnas.2318619121)
Supplement: Supplementary file 1 — Appendix 01 (PDF) [file pnas.2318619121.sapp.pdf]

PNAS

Supporting Information for

The fatty liver disease-causing protein PNPLA3-I148M alters lipid droplet-Golgi dynamics

David J. Sherman, Lei Liu, Jennifer L. Mamrosh, Jiansong Xie, John Ferbas, Brett Lomenick, Mark S. Ladinsky, Rati Verma, Ingrid C. Rulifson, Raymond J. Deshaies

Corresponding author: Raymond J. Deshaies
Email: rdii2003@gmail.com

This PDF file includes:

Supporting Materials and Methods
Figures S1 to S7
Tables S1 to S4
Legends for Movies S1 to S4
Legends for Datasets S1 to S3
SI References

Other supporting materials for this manuscript include the following:

Movies S1 to S4
Datasets S1 to S3

Supporting Materials and Methods

Gene editing and plasmids. Prior to gene editing, cells were grown in antibiotic-free medium. The endogenous *PNPLA3* locus in Hep3B cells was edited using CRISPR to produce a cell line that is homozygous for *PNPLA3-I148M*. First, ribonucleoprotein complexes (PNA Bio) were generated at room temperature for 10 min. ssDNA molecules were subsequently added to the mixture to generate the “master mix” for co-delivery into Hep3B cells (**Table S1**). Cells were washed in PBS and resuspended in Resuspension Buffer R (Invitrogen). Then, cells were mixed 1:1 (v/v) with the master mix for electroporation using a Neon Transfection System (Invitrogen; pulse voltage = 1600, pulse width = 20, pulse number = 1) and immediately transferred to fresh growth medium lacking antibiotics. After growth to a sufficient density, genomic DNA was isolated and editing was confirmed by restriction digestion and droplet digital PCR (Bio-Rad). Cell lines that were edited were clonally selected by limiting dilution. Clonally isolated PNPLA3-I148M cell lines were validated with Sanger sequencing, ddPCR, and next-generation sequencing.

To generate endogenously tagged cell lines, confirmed Hep3B-PNPLA3 (WT) and Hep3B-PNPLA3-I148M clones were edited to insert a 72-bp HA-HiBiT tag at the 3’-end of the endogenous *PNPLA3* locus. Gene editing was performed essentially as described above (**Table S1**). Following electroporation, cells were added to fresh medium lacking antibiotics but containing 20 µM of the NHEJ inhibitor NU7026 (Selleck Chemicals). Following 48-72 h incubation, cells were sorted (BD FACSMelody Cell Sorter) into 96-well plates for clonal selection. Sorting media (pre-filtered) contained 50% FBS, 40% conditioned media (from previous passage), and 10% fresh media supplemented with 10 µM Y-27632 (Sigma) and 1x insulin/transferrin/sodium selenite (ITS; Sigma). Following growth, colonies were selected and screened by TOPO PCR cloning and Sanger sequencing. Clonal identities were further confirmed by NGS-based amplicon sequencing.

pTT5-PNPLA3-His and pTT5-PNPLA3-I148M-His were generated by inserting the coding sequences into the pTT5 vector (1) using Golden Gate Assembly. Briefly, two gBlocks for each construct (Twist Bioscience) were designed with proper overhang sequences using Geneious software (Dotmatics). gBlocks and vector were digested and ligated (in a single reaction) with BsmBI (Thermo Scientific) and T4 DNA ligase (Thermo Scientific). TOP10 cells (Invitrogen) were transformed with the Golden Gate Assembly reactions and colonies were selected on LB-Agar plates containing carbenicillin (Teknova) for Sanger sequencing confirmation (Azenta Life Sciences) using primers pTT5_PNPLA3_fwd, pTT5_PNPLA3_rev, pTT5_PNPLA3_int1, and pTT5_PNPLA3_int2 (**Table S2**).

pTT5-PNPLA3-FLAG and pTT5-PNPLA3-I148M-FLAG were generated using the respective His-tagged construct vectors as templates. Q5 Site-Directed Mutagenesis (NEB) with primers PNPLA3-FLAG_fwd and PNPLA3-FLAG_rev (**Table S2**) was employed to insert the coding sequence for a FLAG tag and STOP codon before the sequence for the His tag.

Allele-specific TaqMan assay. To confirm PNPLA3 variant status (WT vs. I148M) of cell lines and primary hepatocytes, RNA was first isolated by the RNeasy Mini Kit (Qiagen). Samples were then analyzed by digital PCR using a QIAcuity Eight Platform System (Qiagen) with a custom-made allele-specific TaqMan probe ANTZ9CG (ThermoFisher Scientific) that specifically detects PNPLA3 message at the location of the nucleotide change in rs738409 and quantifies reference (WT) copies and mutant variant (I148M) copies. For reference, the pan-PNPLA3 TaqMan probe Hs00228747_m1 (ThermoFisher Scientific) was used. The housekeeping gene probe Hs00427620_m1 (TBP; ThermoFisher Scientific) was employed to normalize expression. Manufacturer’s protocols were followed for dPCR experiments.

Immunoblotting. Unless otherwise specified, gel samples were prepared by boiling for 5 min in 2x SDS sample buffer (125 mM Tris, 4% SDS, 20% glycerol, 0.04% bromophenol blue, pH 6.78) supplemented with 5% β-mercaptoethanol (Sigma). Samples were briefly centrifuged prior to loading onto 4-12% NuPAGE Bis-Tris mini-gels (ThermoFisher Scientific). For HiBiT tag detection, proteins were transferred from the gel onto a 0.45 µm nitrocellulose membrane (ThermoFisher Scientific) at 100 V for 1 h in a Mini Trans-Blot Electrophoretic Transfer Cell (Bio-Rad) kept at 4ºC. For non-HiBiT detection, proteins were transferred onto 0.45 µm polyvinylidene difluoride membranes (ThermoFisher Scientific) using the same conditions. For HiBiT detection, nitrocellulose membranes were washed for 2 x 30 min at room temperature in PBS-0.1% Tween-20 (PBS-T) and then incubated with LgBiT protein overnight at 4ºC, following the protocol of the

Nano-Glo HiBiT Blotting System (Promega). For detection of other proteins, membranes were blocked for 1 h at room temperature in 5% non-fat milk/PBS-T. Membranes were subsequently incubated with primary antibodies overnight at 4ºC (**Table S3**), followed by washes at room temperature (1 x 15 min, 2 x 5 min) and incubation with HRP-conjugated secondary antibodies (Bio-Rad) for 1 h. Following three washes at room temperature, membranes were treated with SuperSignal West Dura Extended Duration Substrate (ThermoFisher Scientific) and affixed to a development cassette. Blots were exposed to BioMax MR film (Carestream Health) in a dark room and developed in an SRX-101A film developer (Konica Minolta).

***In vitro* translation.** Full-length DNA constructs for the coding sequences of human PNPLA3-HA, PNPLA3-I148M-HA, PNPLA3Δ42-62-HA, mouse Ii-op (2), and mouse RAMP4-op (2) were ordered as gBlocks (IDT) with a 5' T7 promoter-linker-Kozak site (5'-TAATACGACTCACTATAGGG AATATTCTTGTTCCCACCATG…). gBlocks were PCR-amplified with Q5 High-Fidelity DNA Polymerase (NEB) using the forward primer T7-Kozak-start-fwd and the reverse primers HA-PolyA-rev (for PNPLA3-HA constructs) or Op-PolyA-rev (for Ii-op and RAMP4-op; **Table S4**). Amplified constructs were used as inputs for reactions (final reaction concentration of 6.96 ng/µL) using the TnT Quick Coupled Transcription/Translation System (Promega). Reactions were performed as described for the TnT system. Briefly, the rabbit reticulocyte lysate master mix was supplemented with EasyTag L-^{35}-Methionine (4% final reaction volume; Perkin Elmer). Canine rough pancreatic microsomes (cRMs; final A_{280} ~ 2.5-3.5), prepared as described (3), were supplemented co-translationally. Reactions were incubated on a benchtop Thermomixer (Eppendorf) at 30ºC for 55 min. To stop the reactions, puromycin dihydrochloride (Gibco) was added (final concentration of 2.5 mM), and samples were placed on ice. For post-translational reactions, cRMs were added following puromycin supplementation, and reactions were incubated at 30ºC for an additional 30 min. Samples (kept on ice) were added to an equal volume of 1x PSB (100 mM KOAc, 2 mM $Mg(OAc)_2$, 50 mM HEPES, pH 7.4) with 250 mM sucrose. Samples were ultracentrifuged in thickwall ultracentrifuge tubes (Thermo Scientific) for 15 min at 200,000 x *g*, 4ºC (Sorvall MTX 150 ultracentrifuge with an S120-AT3 rotor). Supernatants were carefully removed and placed on ice. Pellets were resuspended in 1x PSB with 250 mM sucrose, and then mixed well with 100 mM (final concentration) sodium carbonate, pH 11. Tubes were left on ice for 30 min followed by another round of ultracentrifugation. Supernatants were carefully removed and kept on ice (wash fraction) and pellets were resuspended in PKB (1% SDS, 0.1 M Tris, pH 8.0). 16% of each fraction (initial supernatant, wash, and pellet) was mixed with 2x SDS sample buffer supplemented with 5% β-mercaptoethanol. Samples were boiled for 5 min prior to loading onto NuPAGE 4-12% Bis-Tris gels (Invitrogen). After washing with water and then with 5% glycerol, gels were placed upside down on thick filter paper (Bio-Rad) and covered with saran wrap to dry in a SGD2000 slab gel dryer (ThermoFisher Scientific). Gels were exposed to film and developed as above.

cRM Proteinase K (Lonza) and endoglycosidase H (NEB) digestions were performed as previously described (4, 5). Preparation of trypsin- or mock-treated cRMs has also been described (2).

Preparation of ER-derived microsomes from cells. HEK239T cells (ATCC) were split into 10-cm dishes and grown overnight in DMEM (Gibco) supplemented with 10% FBS. The following day, cells were transfected with pSNAPf-EF1α-PNPLA3-HA-HiBiT (Azenta Life Sciences) using Lipofectamine 3000 and grown for an additional 24 h. Cells were washed once in cold PBS, detached in cold PBS and centrifuged to sediment. Cell pellets were stored at -80ºC until ready for use. Prior to microsome preparation, pellets were thawed on ice, resuspended in 10 mM HEPES-KOH, pH 7.5, and incubated for 10 min on ice. The cells were then pelleted, resuspended in homogenization buffer (10 mM HEPES-KOH, pH 7.5, 10 mM KCl, 1.5 mM $MgCl_2$, 5 mM EGTA, 250 mM sucrose) and passed through a 27 G syringe needle 20 times. The homogenate was subjected to serial centrifugations (600 x *g* for 10 min, 3000 x *g* for 10 min, 100,000 x *g* for 60 min) prior to resuspension in membrane buffer (10 mM HEPES-KOH, pH 7.5, 50 mM KOAc, 2 mM $Mg(OAc)_2$, 1 mM DTT, 250 mM sucrose), as described (5). Proteinase K digestion was done the same way as for cRMs, described above.

Protein production. Proteins were expressed in suspension HEK 293-EBNA1 cells, as described (1, 6). First, complexes of plasmid and PEI MAX (Polysciences) were generated by mixing the appropriate pTT5 expression vector with transfection reagent (ratio of 1 µg vector : 4 µg PEI MAX) in F17 Expression Medium (Gibco). Complexes were incubated for 15 min at room temperature. Plasmid-PEI solutions were added to 293-EBNA1 cells (2 x 10^6 cells/mL) in Expression Medium [F17 Medium supplemented with 0.1% Kolliphor P 188 (Sigma), 6 mM L-glutamine (Gibco), 25 µg/mL G418 Sulfate (Gibco)]. Cells were shaken overnight

at 36ºC, 5% CO_2, 85% humidity. The next day, an equal volume of Expression Medium was added to the culture, and cells were grown in the same incubation conditions for 72 h. Cells were harvested by centrifugation at 3,700 x *g* for 30 min.

For PNPLA3-His constructs, proteins were purified as described (7), except that whole cell lysate (instead of just the membrane fraction) was passed over Ni-NTA. For PNPLA3-FLAG constructs, proteins were purified from the membrane fraction, as described (8).

Lipid binding assays. PIP Strips (Echelon) were blocked overnight in PBS-T with 3% fatty acid-free bovine serum albumin (GoldBio) at 4ºC. The next day, blocking buffer was removed and replaced with blocking buffer supplemented with 2 – 6 µg/mL of purified proteins (PLC-δ1 PH was obtained from Echelon). Strips were incubated on a shaker at room temperature for 1 h, followed by 3 x 10 min washes in PBS-T. Strips were then incubated in blocking buffer containing secondary antibodies (**Table S3**) on a shaker at room temperature for 1 h. Following 3 x 10 min washes, strips were developed with SuperSignal West Dura Extended Duration Substrate and imaged on a ChemiDoc MP Imaging System (Bio-Rad).

Generating LgBiT-expressing cells. Hep3B-HiBiT cells (WT and I148M) were transfected with pCMV-LgBiT (Promega CS1956B03) using FuGENE HD (Promega). After 48 h, cells were treated with Hygromycin to select for incorporation of the plasmid. Stable cell lines were initially tested for bioluminescence using the Nano-Glo Live Cell Assay System (Promega). Bioluminescence was read on an EnVision plate reader (Perkin Elmer).

Live cell bioluminescence imaging. Hep3B-HiBiT-LgBiT cells (WT and I148M), in addition to negative control cells lacking LgBiT, were plated in glass-bottom 96-well plates (Cellvis) and incubated overnight at 37°C, 5% CO_2. Media was replaced with Opti-MEM (Gibco) containing a 1:300 dilution of Nano-Glo Fluorofurimazine Substrate (FFz; Promega), reconstituted per the manufacturer's instructions, 2 µM Hoechst 33342 (ThermoFisher Scientific), and 5 µM BODIPY FL C_5-ceramide (ThermoFisher Scientific). Cells without BODIPY FL C_5-ceramide were included as negative controls. Plates were allowed to equilibrate at 37°C, 5% CO_2 for 20 min on the temperature-controlled stage of the LV200 Bioluminescence Imaging System (Olympus) equipped with an ImagEM X2 EM-CCD camera (C9100-23B; Hamamatsu). Brightfield, bioluminescence, and fluorescence (FITC and DAPI channels) images were captured with a 40x oil-immersion objective. Images were analyzed using cellSens Dimension (Version 3.1) software (Olympus).

Sample preparation for electron microscopy. Cells were grown on 3 mm synthetic sapphire disks (Technotrade International) and treated with 100 µM oleic acid overnight. Cells were then pre-fixed with 3% glutaraldehyde, 1% paraformaldehyde, 5% sucrose in 0.1 M sodium cacodylate. The disks were rinsed with fresh cacodylate buffer containing 10% Ficoll, placed into brass planchettes (Ted Pella, Inc.), and rapidly frozen with an HPM-010 high-pressure freezing machine (Bal-Tec). The frozen samples were transferred under liquid nitrogen to cryotubes (Nunc) containing a frozen solution of 2.5% osmium tetroxide, 0.05% uranyl acetate in acetone. Tubes were loaded into an AFS-2 freeze-substitution machine (Leica Microsystems) and processed at -90°C for 72 h, warmed over 12 h to -20°C, held at that temperature for 6 h, then warmed to 4°C for 2 h. The fixative was removed, and the samples rinsed 4x with cold acetone, after which they were infiltrated with Epon-Araldite resin (Electron Microscopy Sciences) over 48 h. The sapphire disks with affixed cells were flat-embedded onto a Teflon-coated glass microscope slide and covered with a Thermanox coverslip (Electron Microscopy Sciences). Resin was polymerized at 60°C for 48 h and the sapphire disks were excised, leaving the cells as a monolayer within a resin wafer.

Electron microscopy and Dual-Axis tomography. Cells were observed by light microscopy and appropriate regions were extracted with a microsurgical scalpel and glued to the tips of plastic sectioning stubs. Semi-thin (170 nm) serial sections were cut with a UC6 ultramicrotome (Leica Microsystems) using a diamond knife (Diatome). Sections were placed on formvar-coated copper-rhodium slot grids (Electron Microscopy Sciences) and stained with 3% uranyl acetate and lead citrate. Gold beads (10 nm) were placed on both surfaces of the grid to serve as fiducial markers for subsequent image alignment. Sections were placed in a dual-axis tomography holder (Model 2040, E.A. Fischione Instruments) and imaged with a Tecnai T12-G2 transmission electron microscope operating at 120 KeV (ThermoFisher Scientific) equipped with a 2k x 2k CCD camera (XP1000; Gatan, Inc.). Tomographic tilt-series and large-area montaged

overviews were acquired automatically using the SerialEM software package (9, 10). For tomography, samples were tilted ± 62° and images collected at 1° intervals. The grid was then rotated 90° and a similar series taken about the orthogonal axis. Tomographic data was calculated, analyzed, and modeled using the IMOD software package (10, 11) on iMac Pro and Mac Studio M1 computers (Apple, Inc.).

Mass spectrometry. Hep3B cells were grown in 6-well plates (500,000 cells/mL) and washed three times with PBS prior to harvesting. Cell pellets (1 x 10^6 cells/pellet) were flash frozen in liquid nitrogen and stored at -80ºC until ready for analysis. Pellets were thawed on ice and resuspended in 0.05% SDS/0.5 M TEAB. Following multiple rounds of pipetting and brief vortexing, samples were passed through a 23 G syringe needle 30 times on ice and sonicated. Lysates were clarified by centrifugation at 16,000 x g for 10 min at 4ºC. Supernatants were transferred to new tubes and total protein was quantified using a Bradford Assay (Bio-Rad). 20 µg protein per sample was reduced with 3 mM TCEP (Sigma) and alkylated with 10 mM iodoacetamide (Sigma) in the dark. Samples were digested overnight at 37ºC with LysC and trypsin. 5 µg peptides from each sample were labelled using the TMTpro reagents (ThermoFisher Scientific) for 2 hours, followed by quenching with 5% hydroxylamine for 15 minutes, pooling, and drying down. Labelled peptides were mixed and analyzed using two-dimensional liquid chromatography and tandem mass spectrometry, as previously described (12). The pooled samples were desalted followed by phosphopeptide enrichment through sequential use of the High-Select TiO2 and Fe-NTA phosphopeptide enrichment kits (Thermo Scientific). All native peptides not captured by the phosphopeptide enrichment kits were then separated by offline medium pH C4 peptide fractionation (Accucore 150-C4, 2.6um pore size, 150mm X 2.1mm, Thermo Scientific) using gradient mobile phase conditions as previously reported (12). Fractionated peptides were concatenated into 6 pooled fractions, then dried down and stored at −80 °C until mass spectrometry analysis.

Liquid chromatography-mass spectrometry (LC-MS) analysis was carried out on an EASY-nLC 1000 (ThermoFisher Scientific) coupled to an Orbitrap Eclipse Tribrid mass spectrometer (ThermoFisher Scientific). Concatenated native peptide fractions were resuspended in 15 µL 2% acetonitrile, 0.2% formic acid, and 3 – 4 µg peptides per concatenated sample were loaded onto a monolithic column (Capillary EX-Nano MonoCap C18 HighResolution 2000, 0.1 x 2000 mm, Merck) fitted with a silica coated PicoTip emitter (New Objective FS360-20-10-D) and separated over 300 min at a flow rate of 500 nL/min with the following gradient: 2–6% Solvent B (10 min), 6-40% B (260 min), 40-100% B (1 min), and 100% B (29 min). Solvent A consisted of 97.8% water, 2% acetonitrile, and 0.2% formic acid, and solvent B consisted of 19.8% water, 80% acetonitrile, and 0.2% formic acid.

MS1 spectra were acquired in the Orbitrap at 120K resolution with a scan range from 375-2000 m/z, an AGC target of 4e5, and a maximum injection rate of 50 ms in Profile mode. Features were filtered for monoisotopic peaks with a charge state of 2-7 and a minimum intensity of 2.5e4, with dynamic exclusion set to exclude features after 1 time for 60 seconds with a 5-ppm mass tolerance. HCD fragmentation was performed with collision energy of 32% after quadrupole isolation of features using an isolation window of 0.7 m/z, an AGC target of 5e4, and a maximum injection time of 86 ms. MS2 scans were then acquired in the Orbitrap at 50K resolution in Centroid mode with the first mass fixed at 110. Cycle time was set at 1 s.

Phosphopeptide fractions from the TiO2 and Fe-NTA enrichment procedures were each resuspended in 10 µL 2% acetonitrile, 0.2% formic acid, and 8 µL peptides per sample were loaded onto an Aurora 25cm x 75µm ID, 1.6µm C18 reversed phase column (Ion Opticks) and separated over 136 min at a flow rate of 350 nL/min with the following gradient: 2–6% Solvent B (7.5 min), 6-25% B (82.5 min), 25-40% B (30 min), 40-98% B (1 min), and 98% B (15 min). All MS/MS parameters were identical to those for the native peptide runs.

Proteomics data analysis was performed in Proteome Discoverer 2.4 (Thermo Scientific) using the Byonic search algorithm (Protein Metrics) and Uniprot human database. Byonic search parameters for native peptide fractions were as follows: fully Tryptic peptides with no more than 2 missed cleavages, precursor mass tolerance of 10 ppm and fragment mass tolerance of 20 ppm, and a maximum of 3 common modifications and 2 rare modifications. Cysteine carbamidomethylation and TMTpro addition to lysine and peptide N-termini were static modifications. Methionine oxidation and lysine acetylation were common dynamic modifications (up to 2 each). Methionine loss on protein N-termini, methionine loss + acetylation on protein N-termini, protein N-terminal acetylation, and phosphorylation of serine, threonine, and tyrosine were rare dynamic modifications (only 1 each). Percolator FDRs were set at 0.001 (strict) and 0.01 (relaxed). Spectrum file retention time calibration was used with TMTpro addition to peptide N-termini and lysines as static modifications. Reporter ion quantification used a co-isolation threshold of 50 and average

reporter S/N threshold of 10. Normalization was performed on total peptide amount and scaling was performed on all average. Peptide and protein FDRs were set at 0.01 (strict) and 0.05 (relaxed), with peptide confidence at least high, lower confidence peptides excluded, and minimum peptide length set at 6.

Statistical analysis of protein abundances was performed as previously described (13). Protein-level output files from Proteome Discoverer 2.4 were used for analyses. A limma test, implemented in R, was used to determine significance between WT and I148M cell lines (n = 8 per group). *p*-values were corrected for multiple testing by a Benjamini-Hochberg procedure.

For analysis of differential enrichment of biological process keywords in I148M versus WT cell lines, proteins significantly (adj. *p*-value < 0.01) decreased or increased by >1.5 fold in the I148M vs. WT PNPLA3 cell lines were compared with those not significantly changing. Biological process keywords for these sets of proteins were obtained from UniProt and Fisher's exact test was used to determine a *p*-value, which was Bonferroni corrected, for each keyword. Only biological process keywords annotated for at least 5 proteins in at least one of the sets of proteins were considered.

Similar analyses were performed for disease-specific terms. Protein associations for these terms were collected from the Comparative Toxicogenomics Database (CTD; (14)), DisGeNET (15), and (16).

A list of liver-specific genes was obtained from the Tissue-specific Gene Expression and Regulation (TiGER) database.

The mass spectrometry proteomics data have been deposited to the ProteomeXchange Consortium via the PRIDE (17) partner repository with the dataset identifier PXD046335.

RNA-seq. Hep3B cells were grown overnight in 6-well plates (seeded at 500,000 cells/mL). Enough plates were seeded to allow for 5 replicates of the following conditions: WT & I148M basal, WT & I148M 30 min, 1, 2, 4, and 24 h oleic acid (200 µM) treatment. Following the specified treatment, cells (1 x 10^6 cells/well) were washed three times with PBS and lysed in Buffer RLT (Qiagen). Lysates were transferred to Eppendorf tubes on ice and stored at -80ºC until ready for RNA purification. Library preparation from purified RNA, sequencing and analysis protocols have been described previously (18). To determine the changes of gene expression, differential expression analysis between WT and I148M samples at each time point was conducted using DESeq2 (1.26.1) (19). Gene expression fold-change and adjusted *p*-values were generated using default parameters. The RNA-seq data have been deposited in NCBI's Gene Expression Omnibus (20) and are accessible through GEO Series accession number GSE261297.

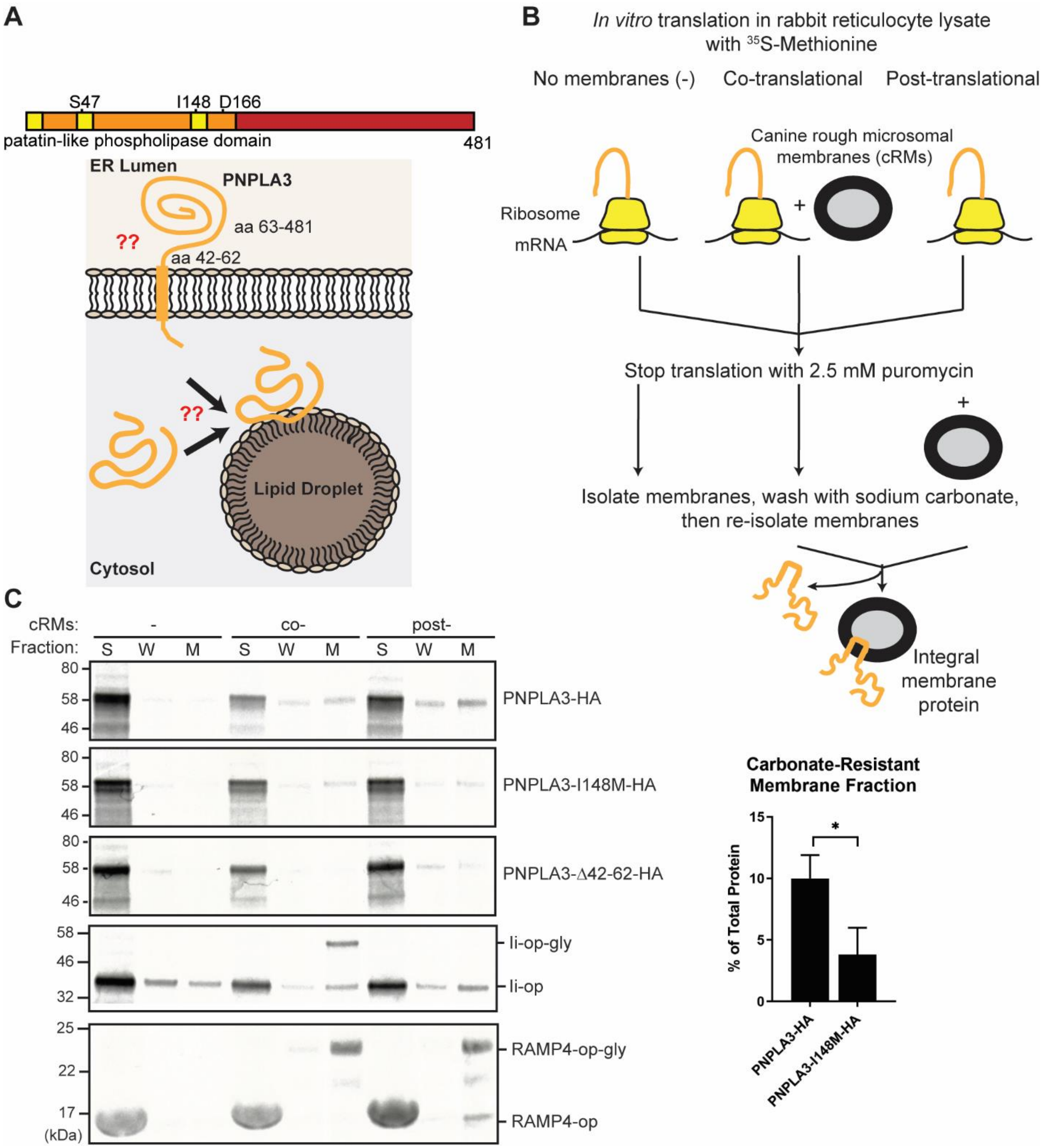

Fig. S1. PNPLA3 lacks an ER-targeting signal sequence. (A) Schematic (top) of the PNPLA3 primary sequence, with predicted hydrophobic segments of roughly ~ 20 amino acids in the PNPLA domain shown in yellow. Cartoon (bottom) depicts contrasting models of where PNPLA3 originates prior to localization on LDs, including a predicted ER topology. Amino acids 42-62 comprise a predicted type II signal anchor sequence. Residues S47 and D166 comprise the putative catalytic dyad. (B) Schematic of *in vitro* translation experimental design. (C) PNPLA3-HA and variants were translated in rabbit reticulocyte lysate in the presence of ^{35}S-methionine. Canine rough microsomal membranes (cRMs) were added co- or post-translationally. Membranes were isolated, washed with sodium carbonate, then re-isolated. As controls for membrane insertion, the signal recognition particle-dependent type II signal anchor sequence-containing protein invariant chain (Ii) and the tail-anchored protein RAMP4, both containing C-terminal opsin (op) tags,

were also translated. “S” = soluble fraction; “W” = carbonate wash; “M” = membrane fraction. Quantification of PNPLA3 in membrane fractions (right). * $p < 0.05$, determined by Student’s t-test (n = 3).

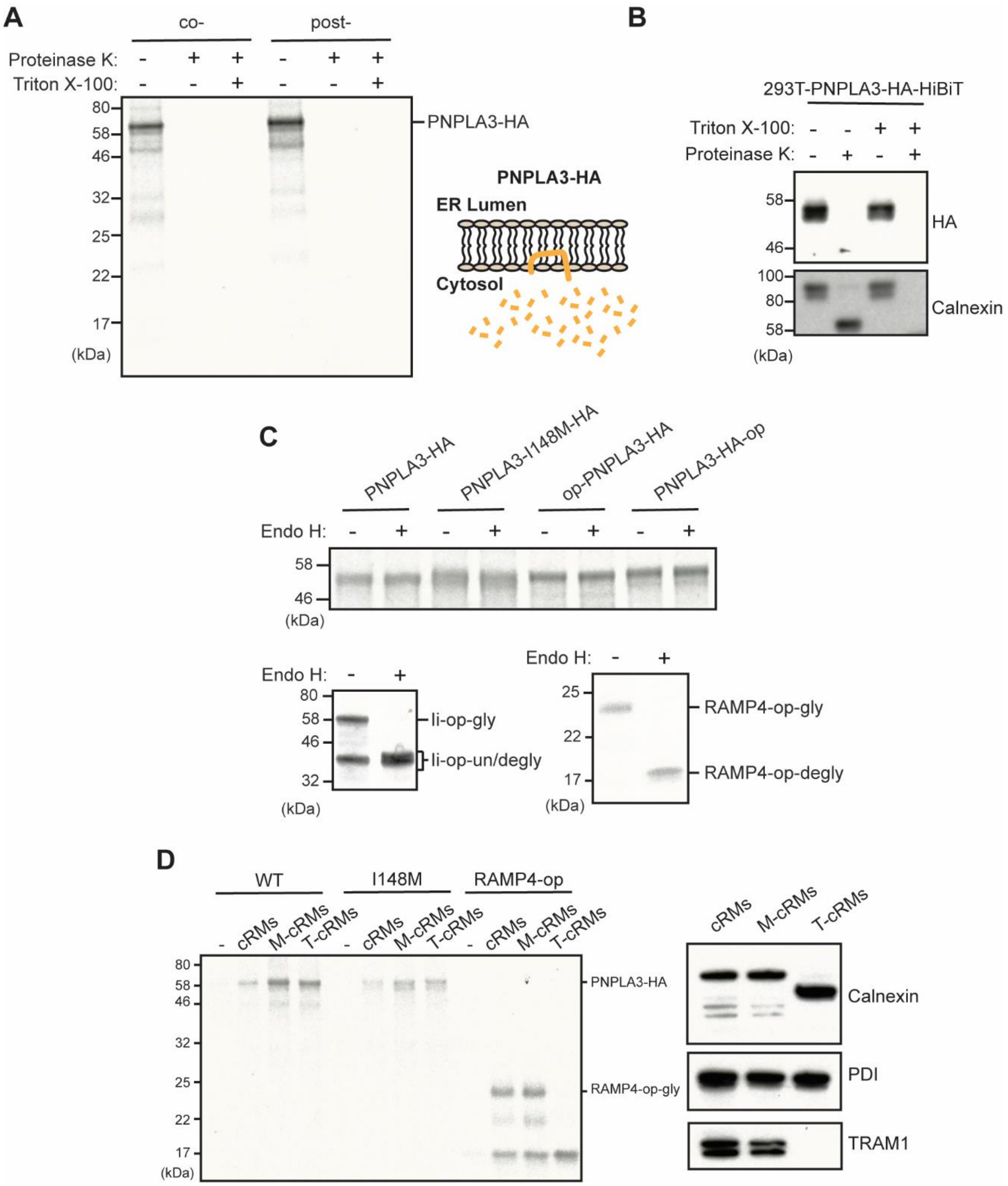

Figure S2. Membrane-bound PNPLA3 is cytosolically exposed. (A) cRMs added co- or post-translationally were incubated with Proteinase K with or without Triton X-100. (B) HEK293T cells were transiently transfected with a PNPLA3-HA-HiBiT vector. After 48 h, microsomes were isolated from the cells and subsequently treated with Proteinase K with or without Triton X-100. (C) cRM's added post-translationally were solublized and treated with Endoglycosidase H. Control reactions were done with Ii-op and RAMP4-op. (D) cRM's were mock-treated (M-cRMs) or trypsin-treated (T-cRMs) to remove exposed portions of membrane proteins. These cRMs were added post-translationally to translation reactions. Following a carbonate wash and re-isolation of cRMs, localization of PNPLA3-HA constructs or RAMP4-op were assessed by SDS-PAGE (left). cRMs were characterized by immunonlotting to ensure trypsin digestion of exposed proteins (right).

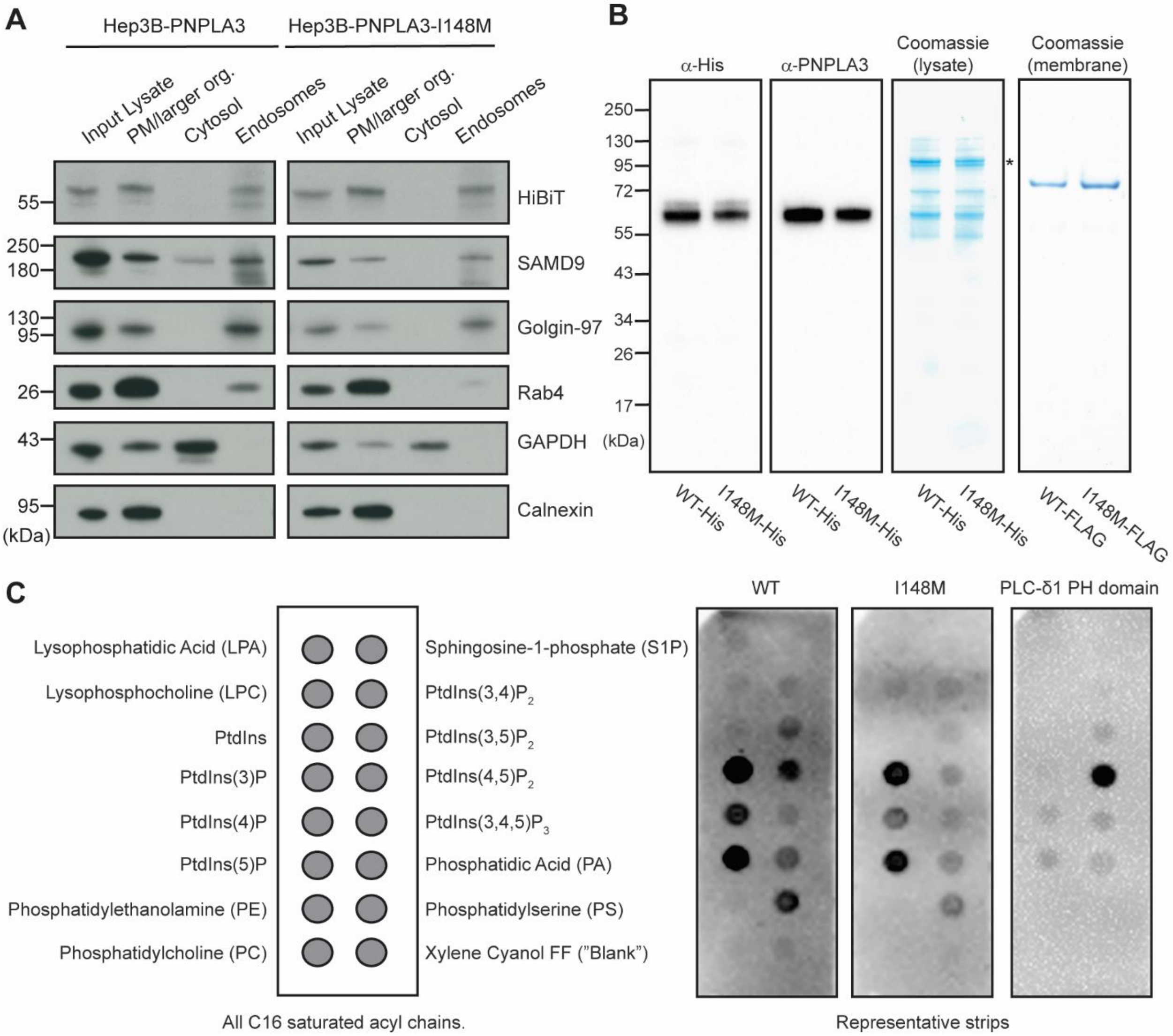

Fig. S3. PNPLA3-I148M fractionates with endosomes and interacts with membrane phosphoinositides. (A) Endogenous PNPLA3-HiBiT and PNPLA3-I148M-HiBiT are enriched in the plasma membrane (PM)/larger organelle fraction and the endosomal fraction. (B) Immunoblotting and Coomassie staining of purified PNPLA3 constructs. *SDS-resistant PNPLA3 dimers. (C) Purified PNPLA3-His, PNPLA3-I148M-His, and GST-PLC-δ1 PH domain were incubated with membranes spotted with 100 pmol of different lipids (left). After washing the membranes thoroughly, they were incubated with anti-His or anti-GST secondary antibodies (HRP-conjugated) and imaged using chemiluminescence. Experiment was repeated at least 3 times for each protein, with representative images shown (right). Experiment was repeated with PNPLA3-FLAG constructs, with similar results.

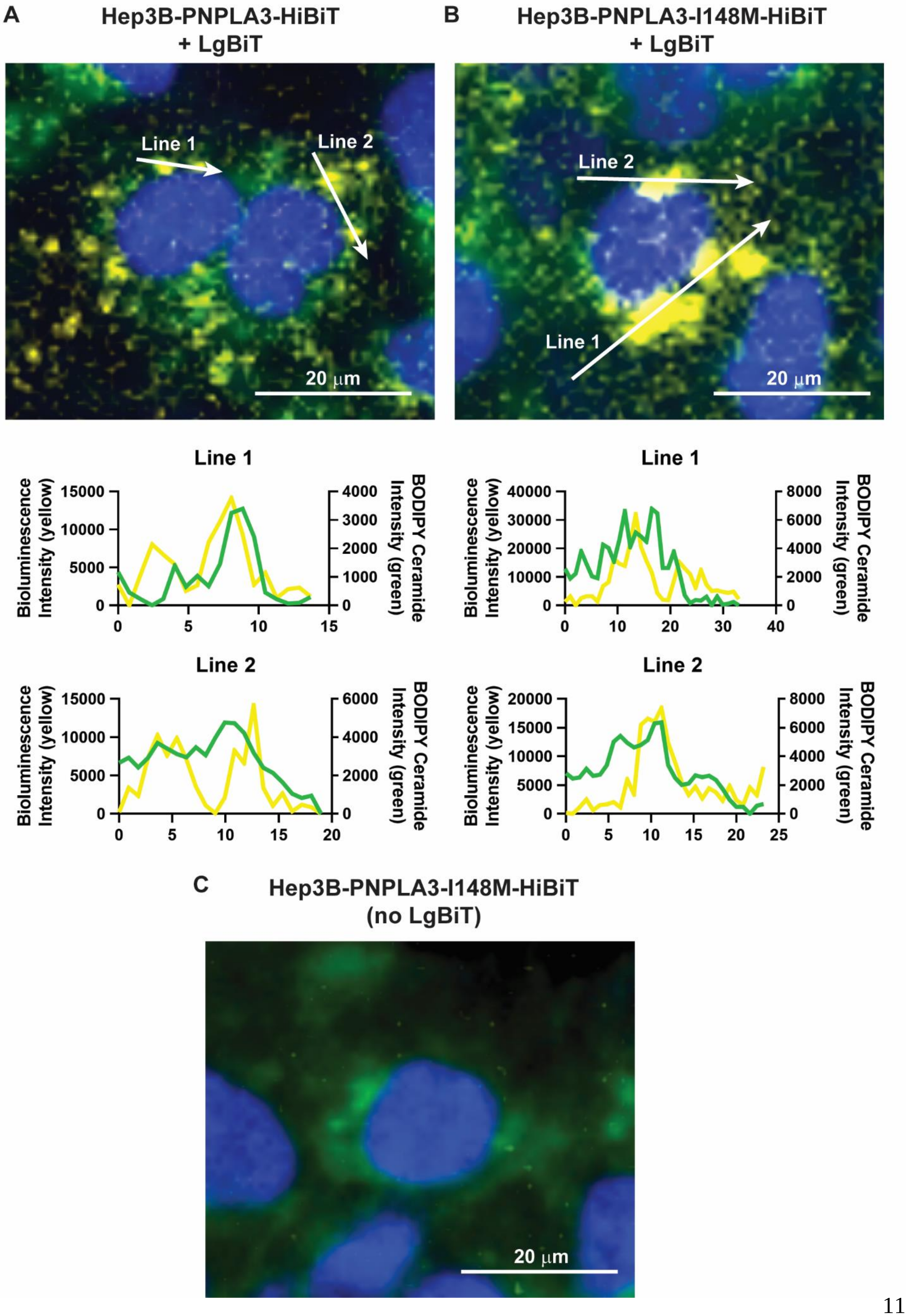
A
Hep3B-PNPLA3-HiBiT
+ LgBiT
Line 1
Line 2
20 μm
B
Hep3B-PNPLA3-I148M-HiBiT
+ LgBiT
Line 2
Line 1
20 μm
Line 1
Bioluminescence Intensity (yellow)
BODIPY Ceramide Intensity (green)
15000
10000
5000
0
4000
3000
2000
1000
0
0
5
10
15
Line 1
40000
30000
20000
10000
0
8000
6000
4000
2000
0
0
10
20
30
40
Line 2
15000
10000
5000
0
6000
4000
2000
0
0
5
10
15
20
Line 2
20000
15000
10000
5000
0
8000
6000
4000
2000
0
0
5
10
15
20
25
C
Hep3B-PNPLA3-I148M-HiBiT
(no LgBiT)
20 μm

Fig. S4. PNPLA3 and PNPLA3-I148M co-localize with the Golgi in live cells. Representative live-cell bioluminescence images of (A) Hep3B-PNPLA3-HiBiT and (B) Hep3B-PNPLA3-I148M-HiBiT cells co-expressing LgBiT, and (C) Hep3B-PNPLA3-I148M-HiBiT cells without LgBiT expression. Cells were treated with a fluorofurimazine (FFz) bioluminescence substrate (yellow stain), BODIPY FL C_5-ceramide (for fluorescence labeling of the Golgi; green stain), and Hoechst 33342 (for fluorescence labeling of the nuclei; blue stain). Images were captured using the Olympus LV200 Bioluminescence Imaging System. Representative line intensity profiles of bioluminescence and green fluorescence were generated using Olympus cellSens Dimension (Version 3.1) software to show co-localization of PNPLA3-HiBiT and PNPLA3-I148M-HiBiT with the Golgi.

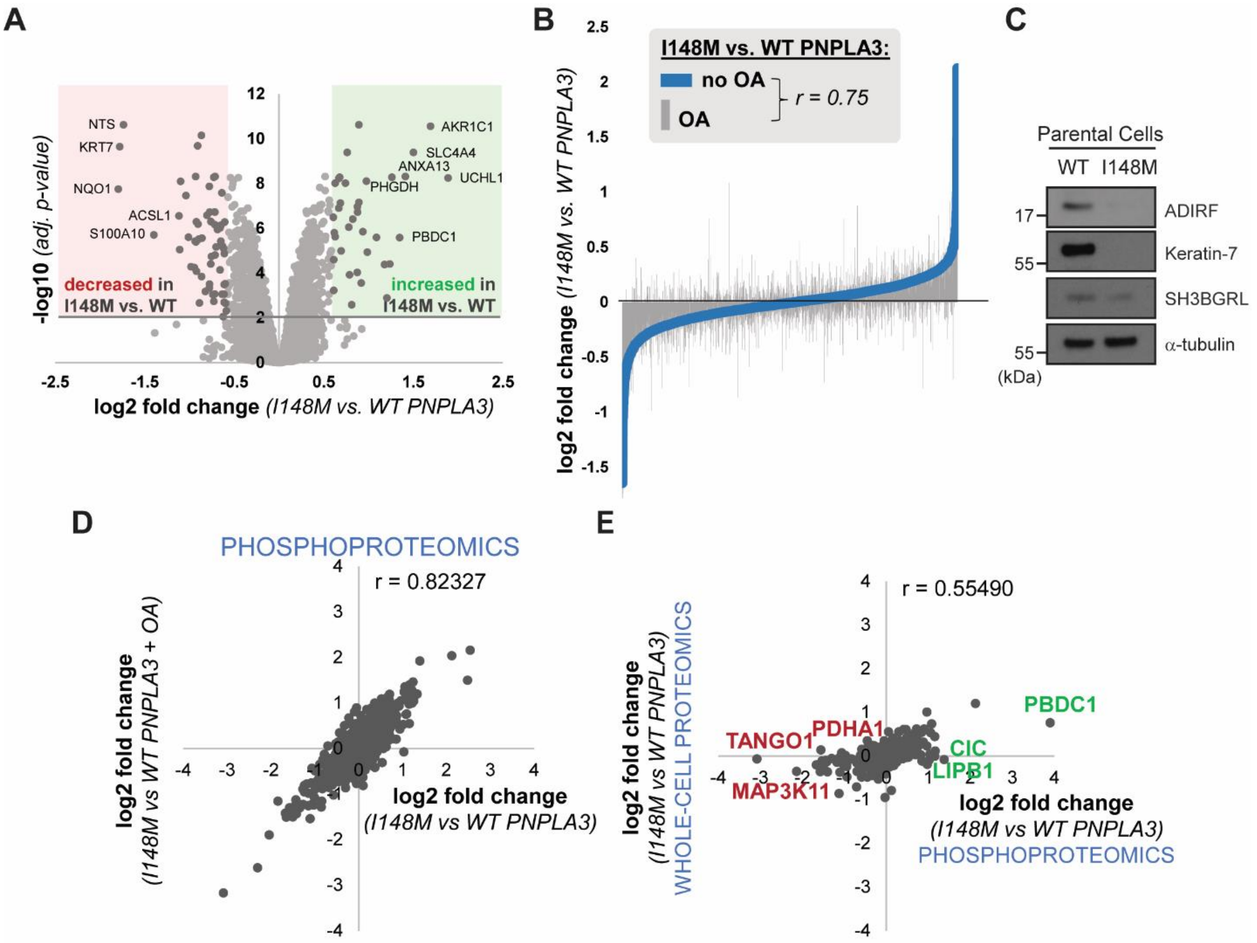

F

Source	Disease	# Terms
Comparative Toxicogenomics Database (CTD)	fatty liver (MESH:D005234)	1853
Comparative Toxicogenomics Database (CTD)	hepatocellular carcinoma (MESH:D006528)	1132
DisGeNET	fatty liver disease (CUI: C4529962)	741
DisGeNET	liver fibrosis (CUI: C0239946)	1179
DisGeNET	adult hepatocellular carcinoma (CUI: C0279607)	150
Ref. 16	liver-enriched genes in hepatocellular carcinoma	188

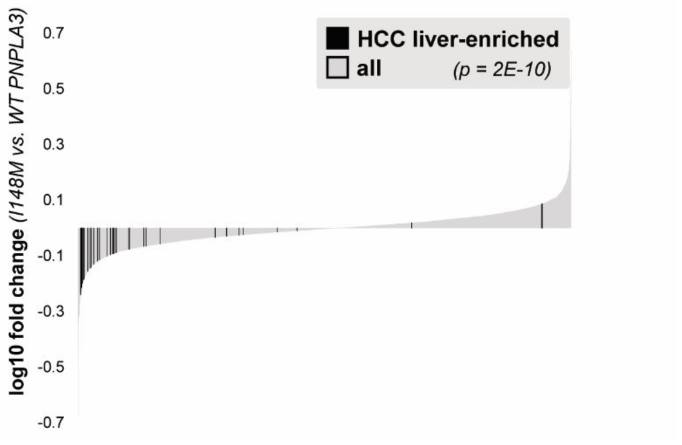

Fig. S5. PNPLA3-I148M has a distinct effect on proteomic and transcriptomic cellular changes relative to oleic acid treatment. (A) Volcano plot of proteins quantified by mass spectrometry in cells expressing WT or PNPLA3-I148M, following 16 h treatment with 200 µM oleic acid. Red and green compartments contain proteins significantly (adj. *p*-value < 0.01) decreased or increased, respectively, by >1.5 fold in the I148M vs. WT cell lines. (B) Comparison of the log2 fold change (I148M vs. WT PNPLA3) between two mass spectrometry experiments, in which cells were treated with or without oleic acid for 16 h. (C) Confirmation of top proteomic hits (downregulated in I148M; see Fig. 3A) in parental Hep3B cells. (D) Scatterplot of phosphopeptides quantified by mass spectrometry in vehicle- (2313 peptides) or oleic

acid- (OA; 2086 peptides) treated Hep3B-HiBiT cells expressing WT or PNPLA3-I148M. The Pearson correlation coefficient between vehicle and oleic acid-treated groups is shown. (E) Phosphopeptides from vehicle-treated cells expressing WT or PNPLA3-I148M were filtered to include only those with one defined phosphorylation site. Scatterplot depicts log2 PNPLA3-I148M vs. WT PNPLA3 fold changes from this experiment compared with log2 PNPLA3-I148M vs. WT PNPLA3 fold changes from the whole-cell proteomics experiment (see Fig. 3A). The Pearson correlation coefficient between these two independent experiments (635 peptides and 421 proteins) is shown. The top 3 peptides differentially (green = increased; red = decreased) phosphorylated are labeled. (F) Sources of liver disease-related gene sets. (G) Plot showing log10 fold change for proteins quantified in the I148M vs. WT PNPLA3 untreated cell lines. Those reported to be tissue-enriched genes in hepatocellular carcinoma (HCC) are shown in black (16). Kolmogorov-Smirnov *p*-value testing for a difference in fold change distribution between the two protein sets is reported.

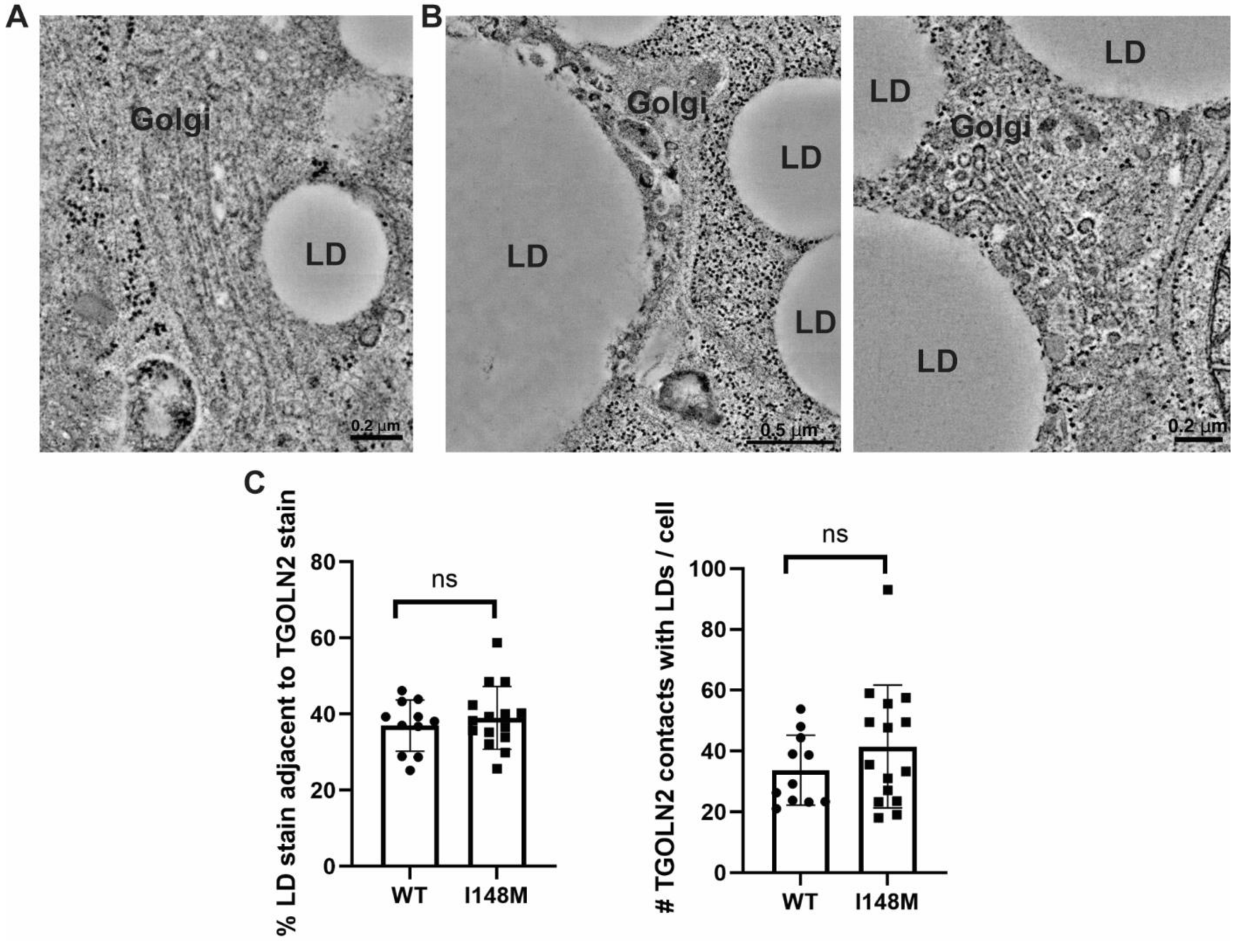

Fig. S6. LD-Golgi contacts in Hep3B cells. Representative TEM images from WT (A) and I148M (B) Hep3B cells pre-treated with 100 µM oleic acid for 16 h. (C) Hep3B cells expressing endogenous PNPLA3 or PNPLA3-I148M were treated with 100 µM oleic acid for 16 h, fixed and stained with LipidTOX Green to label LDs and anti-TGOLN2 to label the *trans*-Golgi network. Lipid droplet-TGOLN2 contact sites in ~ 45 cells per cell line were quantified using Imaris software. *p*-values were calculated from Student's *t*-tests. ns $p > 0.05$.

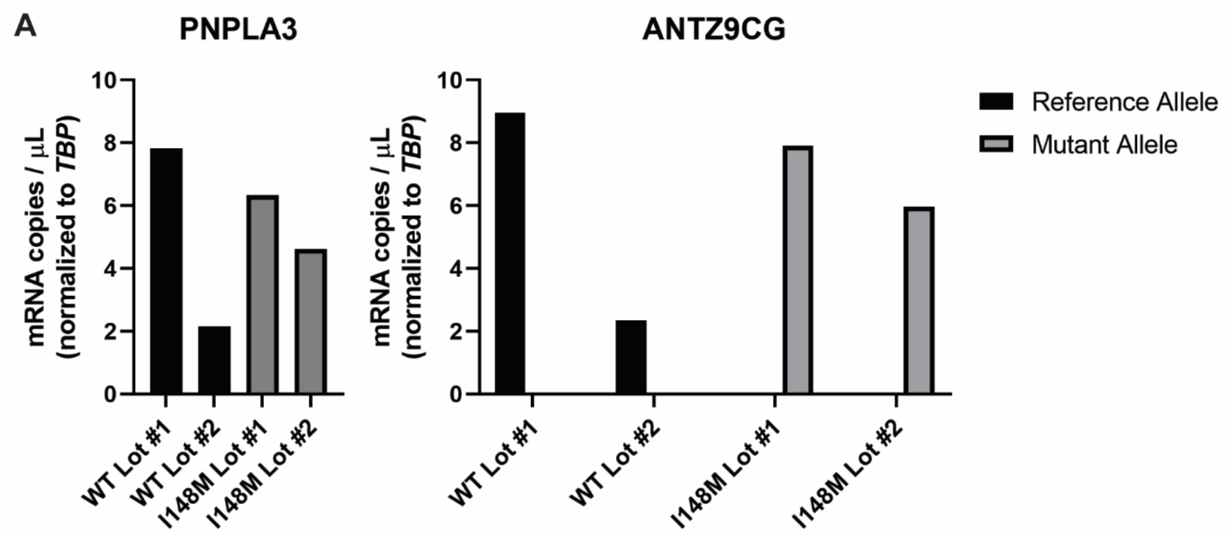

B

Identifier	Vendor	Lot	BMI	Age at Death
WT #1	Lonza	HUM4111C	31.6	27
WT #2	Lonza	HUM4056B	28.9	55
I148M #1	BioIVT	FZQ	27.4	69
I148M #2	BioIVT	IXL	25.0	56

Fig. S7. Identification of primary human hepatocyte lots that express PNPLA3-I148M. (A) Individual lots of primary human hepatocytes were assessed for *PNPLA3* expression by digital PCR using a TaqMan pan-PNPLA3 primer/probe set (left) and a custom TaqMan allele-specific primer/probe set (ANTZ9CG; right) that can distinguish the reference and mutant *PNPLA3* alleles. (B) Table showing key identifying information about primary human hepatocyte lots.

Table S1. sgRNA and ssDNA for Hep3B gene editing.

Name	Description	5'-3' Sequence
sgRNA-1	For generation of *PNPLA3-I148M*	TGCTTCATCCCCTTCTACAG
sgRNA-2	For generation of HA-HiBiT tag at 3' end	AGTCTGTGAGTCACTTGAGG
sgRNA-3	For generation of HA-HiBiT tag at 3' end	AAGAGTCTGTGAGTCACTTG
ssDNA-1	For generation of *PNPLA3-I148M*	CTTATGAAGGATCAGGAAAATTAAAAGGGTGCTCTCGC CTATAACTTCTCTCTCCTTTGCTTTCACAGGCCTTGGTA TGTTCCTGCTTCATGCCCTTCTACAGTGGCCTTATCCC TCCTTCCTTCAGA
ssDNA-2	For generation of HA-HiBiT tag at 3' end	TAAAGTACCTGCTGGTGCTGAGGGGCTCTCCACCTTT CCCAGTTTTTCACTAGAGAAGAGTCTGTACCCATACGA TGTTCCAGATTACGCTGGGAGCAGCGGGGTGAGCGG CTGGCGGCTGTTCAAGAAGATTAGCTGAGTCACTTGA GGAGGCGAGTCTAGCAGATTCTTTCAGAGGTGCTAAA GTTTCCCATCTTTGT

Table S2. Sequencing and Mutagenesis Primers.

Primer Name	5'-3' Sequence	Description
pTT5_PNPLA3_fwd	CGAGGAGGATTTGATATTC	Sequencing pTT5-PNPLA3-His constructs
pTT5_PNPLA3_rev	CTTCCGAGTGAGAGACAC	Sequencing pTT5-PNPLA3-His constructs
pTT5_PNPLA3_int1	AAGCAAGTTCCTCCGACAGG	Sequencing pTT5-PNPLA3-His constructs
pTT5_PNPLA3_int2	AAGCTCAGTCTACGCCTCTG	Sequencing pTT5-PNPLA3-His constructs
PNPLA3_FLAG_fwd	CGACGATAAGTAGCACCATCA CCATCACCATCACCATTAG	Mutagenesis to make pTT5-PNPLA3-FLAG
PNPLA3_FLAG_rev	TCATCCTTGTAATCCCCGCTG CTCCCCAGACT	Mutagenesis to make pTT5-PNPLA3-FLAG

Table S3. Antibodies.

Antibody	Vendor	Catalog #	Use
β-actin	Cell Signaling	4970	Immunoblotting
GAPDH	EMD Millipore	MAB374	Immunoblotting
α-tubulin	Cell Signaling	2144	Immunoblotting
PLIN2	Cell Signaling	95109	Immunoblotting
Calnexin	Cell Signaling	2679	Immunoblotting
PERK	Cell Signaling	3192	Immunoblotting
TGN46	Thermo Scientific	MA532523	Immunoblotting
GM130	Cell Signaling	12480	Immunoblotting
Golgin-97	Cell Signaling	13192	Immunoblotting
SAMD9	Sigma	HPA021319	Immunoblotting
RAB4	Cell Signaling	2167	Immunoblotting
COX IV	Cell Signaling	4850	Immunoblotting
GLUT1	Cell Signaling	12939	Immunoblotting
TGOLN2	Sigma	HPA012723	Immunofluorescence
UCHL1	Cell Signaling	13179	Immunoblotting
AKR1C1	Thermo Scientific	PA521672	Immunoblotting
PHGDH	Cell Signaling	66350	Immunoblotting
APM2 (ADIRF)	Thermo Scientific	PA112909	Immunoblotting
Keratin 7	Cell Signaling	4465	Immunoblotting
SH3BGRL	Thermo Scientific	MA527200	Immunoblotting
PDI	Enzo	ADISPA890D	Immunoblotting
TRAM1	Thermo Scientific	PA521955	Immunoblotting
HA-tag	Thermo Scientific	26183	Immunoblotting
Goat Anti-Rabbit IgG-HRP	Bio-Rad	1706515	Immunoblotting secondary Ab
Goat Anti-Mouse IgG-HRP	Bio-Rad	1706516	Immunoblotting secondary Ab
Anti-His-HRP	Thermo Scientific	MA121315HRP	Lipid binding assay
Anti-GST-HRP	Thermo Scientific	MA4004HRP	Lipid binding assay
Goat Anti-Rabbit IgG-AlexaFluor Plus 647	Thermo Scientific	A32733	Immunofluorescence secondary Ab

Table S4. Primers for *in vitro* translation.

Primer Name	5'-3' Sequence
T7-Kozak-start-fwd	GAAATATAAGTAATACGACTCACTATAGGGAATATTCTTGTTCCCACCATG
HA-PolyA-rev	AATTTTTTTTTTTTTTCAGGCGTAGTCGGGCACAT
Op-PolyA-rev	AATTTTTTTTTTTTTTCAATCCACGGTTTTGTTGCTAAACGG

Movie S1 (separate file). Nanolive time course for untreated Hep3B PNPLA3 (WT) cells.

Movie S2 (separate file). Nanolive time course for untreated Hep3B PNPLA3-I148M cells.

Movie S3 (separate file). Nanolive time course for oleic acid-treated Hep3B PNPLA3 (WT) cells.

Movie S4 (separate file). Nanolive time course for oleic acid-treated Hep3B PNPLA3-I148M cells.

Dataset S1 (separate file). Proteomics dataset for untreated Hep3B cells (WT and I148M).

Dataset S2 (separate file). Proteomics dataset for oleic acid-treated Hep3B cells (WT and I148M).

Dataset S3 (separate file). Phosphoproteomics dataset for untreated and oleic acid-treated Hep3B cells (WT and I148M).

SI References

1. Y. Durocher, S. Perret, A. Kamen, High-level and high-throughput recombinant protein production by transient transfection of suspension-growing human 293-EBNA1 cells. *Nucleic Acids Res* **30**, E9 (2002).
2. B. Schrul, R. R. Kopito, Peroxin-dependent targeting of a lipid-droplet-destined membrane protein to ER subdomains. *Nat Cell Biol* **18**, 740-751 (2016).
3. P. Walter, G. Blobel, Preparation of microsomal membranes for cotranslational protein translocation. *Methods Enzymol* **96**, 84-93 (1983).
4. A. Sharma, M. Mariappan, S. Appathurai, R. S. Hegde, In vitro dissection of protein translocation into the mammalian endoplasmic reticulum. *Methods Mol Biol* **619**, 339-363 (2010).
5. S. K. Radhakrishnan, W. den Besten, R. J. Deshaies, p97-dependent retrotranslocation and proteolytic processing govern formation of active Nrf1 upon proteasome inhibition. *Elife* **3**, e01856 (2014).
6. V. Jager *et al.*, High level transient production of recombinant antibodies and antibody fusion proteins in HEK293 cells. *BMC Biotechnol* **13**, 52 (2013).
7. P. Pingitore *et al.*, Recombinant PNPLA3 protein shows triglyceride hydrolase activity and its I148M mutation results in loss of function. *Biochim Biophys Acta* **1841**, 574-580 (2014).
8. Y. Huang, J. C. Cohen, H. H. Hobbs, Expression and characterization of a PNPLA3 protein isoform (I148M) associated with nonalcoholic fatty liver disease. *J Biol Chem* **286**, 37085-37093 (2011).
9. D. N. Mastronarde, Automated electron microscope tomography using robust prediction of specimen movements. *J Struct Biol* **152**, 36-51 (2005).
10. D. N. Mastronarde, Correction for non-perpendicularity of beam and tilt axis in tomographic reconstructions with the IMOD package. *J Microsc* **230**, 212-217 (2008).
11. J. R. Kremer, D. N. Mastronarde, J. R. McIntosh, Computer visualization of three-dimensional image data using IMOD. *J Struct Biol* **116**, 71-76 (1996).
12. A. Manousopoulou *et al.*, Quantitative proteomic profiling of primary cancer-associated fibroblasts in oesophageal adenocarcinoma. *Br J Cancer* **118**, 1200-1207 (2018).
13. C. S. Shin *et al.*, LONP1 and mtHSP70 cooperate to promote mitochondrial protein folding. *Nat Commun* **12**, 265 (2021).
14. A. P. Davis *et al.*, Comparative Toxicogenomics Database (CTD): update 2023. *Nucleic Acids Res* **51**, D1257-D1262 (2023).
15. J. Pinero *et al.*, The DisGeNET knowledge platform for disease genomics: 2019 update. *Nucleic Acids Res* **48**, D845-D855 (2020).
16. B. Li *et al.*, Liver-enriched Genes are Associated with the Prognosis of Patients with Hepatocellular Carcinoma. *Sci Rep* **8**, 11197 (2018).

17. Y. Perez-Riverol *et al.*, The PRIDE database resources in 2022: a hub for mass spectrometry-based proteomics evidences. *Nucleic Acids Res* **50**, D543-D552 (2022).
18. M. J. Giffin *et al.*, AMG 757, a Half-Life Extended, DLL3-Targeted Bispecific T-Cell Engager, Shows High Potency and Sensitivity in Preclinical Models of Small-Cell Lung Cancer. *Clin Cancer Res* **27**, 1526-1537 (2021).
19. M. I. Love, W. Huber, S. Anders, Moderated estimation of fold change and dispersion for RNA-seq data with DESeq2. *Genome Biol* **15**, 550 (2014).
20. R. Edgar, M. Domrachev, A. E. Lash, Gene Expression Omnibus: NCBI gene expression and hybridization array data repository. *Nucleic Acids Res* **30**, 207-210 (2002).
